# Unravelling the Distribution of Secondary Metabolites in *Olea europaea* L.: Exhaustive Characterization of Eight Olive-Tree Derived Matrices by Complementary Platforms (LC-ESI/APCI-MS and GC-APCI-MS)

**DOI:** 10.3390/molecules23102419

**Published:** 2018-09-20

**Authors:** Lucía Olmo-García, Nikolas Kessler, Heiko Neuweger, Karin Wendt, José María Olmo-Peinado, Alberto Fernández-Gutiérrez, Carsten Baessmann, Alegría Carrasco-Pancorbo

**Affiliations:** 1 Department of Analytical Chemistry, Faculty of Science, University of Granada, Ave. Fuentenueva s/n, 18071 Granada, Spain; luciaolmo@ugr.es (L.O.-G.); albertof@ugr.es (A.F.-G.); 2 Bruker Daltonik GmbH, Fahrenheitstraße 4, 28359 Bremen, Germany; Nikolas.Kessler@bruker.com (N.K.); Heiko.Neuweger@bruker.com (H.N.); Karin.Wendt@bruker.com (K.W.); Carsten.Baessmann@bruker.com (C.B.); 3 Acer Campestres S.L. Almendro, 37 (Pol. Ind. El Cerezo), Castillo de Locubín, 23670 Jaén, Spain; j.olmo@elayo.com

**Keywords:** *Olea europaea* L., liquid chromatography, gas chromatography, mass spectrometry, secondary metabolites

## Abstract

In order to understand the distribution of the main secondary metabolites found in *Olea europaea* L., eight different samples (olive leaf, stem, seed, fruit skin and pulp, as well as virgin olive oil, olive oil obtained from stoned and dehydrated fruits and olive seed oil) coming from a Picudo cv. olive tree were analyzed. All the experimental conditions were selected so as to assure the maximum coverage of the metabolome of the samples under study within a single run. The use of LC and GC with high resolution MS (through different ionization sources, ESI and APCI) and the annotation strategies within MetaboScape 3.0 software allowed the identification of around 150 compounds in the profiles, showing great complementarity between the evaluated methodologies. The identified metabolites belonged to different chemical classes: triterpenic acids and dialcohols, tocopherols, sterols, free fatty acids, and several sub-types of phenolic compounds. The suitability of each platform and polarity (negative and positive) to determine each family of metabolites was evaluated in-depth, finding, for instance, that LC-ESI-MS (+) was the most efficient choice to ionize phenolic acids, secoiridoids, flavonoids and lignans and LC-APCI-MS was very appropriate for pentacyclic triterpenic acids (MS (−)) and sterols and tocopherols (MS (+)). Afterwards, a semi-quantitative comparison of the selected matrices was carried out, establishing their typical features (e.g., fruit skin was pointed out as the matrix with the highest relative amounts of phenolic acids, triterpenic compounds and hydroxylated fatty acids, and seed oil was distinctive for its high relative levels of acetoxypinoresinol and tocopherols).

## 1. Introduction

Olive tree (*Olea europaea* L.), which has accompanied mankind since prehistoric times, has played a fundamental role in the economic, social, and cultural spheres of Mediterranean civilizations [1,2]. Nowadays, along with the consumption of olives and olive oil in the diet, the use of different olive fractions with therapeutic purposes is still deeply rooted in traditional medicine from many parts of the world. It is now known that some of these traditional usages are supported by scientific evidences. In fact, different in vitro and in vivo studies carried out on plant materials or isolated components from olive tree and virgin olive oil (VOO) have demonstrated their health-promoting effects against inflammatory and age-dependent ailments, such as cardiovascular and neurodegenerative diseases, diabetes or cancer, among others [3,4,5,6,7]. The phytochemical characterization of these matrices has revealed the presence of a plethora of bioactive secondary metabolites belonging to different chemical classes, mainly phenolic and triterpenic compounds, tocopherols, sterols, and pigments [3,4]. Some of these phytonutrients found in olive fruits are transferred into the VOO [8,9,10] and are considered to be mainly responsible for the healthy benefits derived from its consumption [11,12]. Logically, the rest of them remain in the VOO processing by-products, which have been pointed out as very valuable sources of bioactive compounds [13,14,15]. Both effluents (olive mill wastewater) and solid wastes (olive pomace) containing phenolic compounds, organic acids, and lipids, are harmful to the environment. Consequently, some of the current VOO by-products management practices involve bioconversion to reduce their environmental impact or the recovery of those phytochemicals with potential applications in food, pharmaceutical and cosmetic industries [16,17,18]. Olive leaves (either coming from pruning or harvested olive fruits washing in table olive or VOO industries) represent the other major olive tree derived by-product and are also very rich in valuable metabolites; different reviews addressing olive leaf characterization, extraction techniques and applications can be found in literature [19,20]. Olive stones from pitted olive table industry have also been identified as a source of proteins and phenols with industrial applications [21].

The transformation of olive fruit and the valorization of by-products are currently considered as parts of the same integral cycle of olive grove exploitation. New environmentally friendly extraction techniques of high value-added compounds from olive derived residues are emerging as a way to increase the profitability of olive sector [22]. Moreover, formerly unexplored products such as olive seed oil and novel processing methods are being investigated in an attempt to take advantage of all olive tree derived matrices with zero waste generation [23,24,25]. It is clear that the exhaustive characterization of every olive tree fraction (olive fruit organs and resulting oils, as well as leaves and stems) is crucial when looking for new applications or new sources of bioactive compounds [26].

Different metabolomic approaches, mainly based on nuclear magnetic resonance (NMR) [27,28] and mass spectrometry (MS) [29], have been applied to the study of small metabolites in olive tree matrices. The use of separative techniques such as liquid (LC) or gas (GC) chromatography prior to MS detection is commonplace when analyzing complex plant derived samples [30]. Both LC/GC-MS based metabolic profiling approaches (primarily focused on the polar phenols fraction) have been used to study olive plant organs (leaves, stems, wood, roots) [31,32,33], olive fruits [34,35], and VOO [36,37]. Different coupling interfaces can be used depending on the physicochemical properties of the analytes under study [38]. Electrospray ionization (ESI) and atmospheric pressure chemical ionization (APCI) sources are the most frequently chosen for LC-MS analyses. Both of them can offer complementary information; for instance, whereas ESI has been the most commonly used interface for phenols profiling [33,34,36], APCI has demonstrated some advantages for the detection of specific families of compounds such as tocopherols or sterols and has also proved to be suitable for phenolic compounds determination [39,40,41]. In GC-MS, electron impact (EI) is the most used ionization source because, at 70 eV, it produces a characteristic fragmentation pattern that enables identification of compounds by means of mass spectral library search. However, the use of softer ionization techniques such as CI or APCI, which can preserve the pseudo-molecular ion information, is becoming increasingly popular since they allow the identification of unknown compounds missing in commercial libraries [42]. 

In this study, multi-analyte methods were applied to the metabolic profiling of eight matrices coming from a Picudo cv. olive tree, including plant materials (leaves, stems and fruit epicarp, mesocarp and seed) and oils (VOO, olive oil obtained from stoned and dehydrated fruits and olive seed oil). Sample preparation consisted in the application of a very unselective protocol aiming at the extraction of as many compounds as possible. The resulting extracts were analyzed by LC-QTOF-MS (coupled through two kinds of interfaces, ESI and APCI) and GC-APCI-QTOF-MS (after derivatization of the prepared extracts) in order to compare the analytical performance of each platform and maximize the achieved information. Our final goal was to understand the distribution of the detected compounds on the studied matrices.

## 2. Results and Discussion

### 2.1. Comprehensive Qualitative Determination of the Matrices under Study

In a first stage of the study, 50 standards or isolated fractions of compounds already detected in *Olea europaea* L. matrices were analyzed by GC-APCI-MS, LC-ESI-MS, and LC-APCI-MS, in order to create an analyte list with the *m*/*z* and retention time (Rt) of known molecules which could help to achieve the identification of as many compounds as possible in the selected samples. Afterwards, all the prepared extracts were analyzed by using the three described methodologies. LC-MS analyses were conducted at least 4 times with each interface (ESI and APCI), in positive and negative polarities, both in normal MS and auto MS/MS modes. Figure S1 (Supplementary Materials) shows typical chromatograms obtained with each platform and polarity when the olive oil obtained from stoned and dehydrated fruits is analyzed.

All the acquired files were imported into MetaboScape. Apart from selecting the optimal threshold for features selection depending on the intensity of the obtained chromatograms, the choice of the target ions was carefully optimized in order to correctly detect potential adducts or fragments belonging to each compound. When using negative polarity in LC-MS analyses, the pseudo-molecular ion [M − H]^−^ was the major signal found in the spectra regardless of the interface. On the contrary, in positive ion mode, [M + H]^+^ was not the prevalent MS signal in many cases; water losses were very common ([M − H_2_O + H]^+^) and alkali adducts (mainly [M + Na]^+^ and [M + K]^+^) were also frequently found, especially with the ESI source. Regarding GC-APCI-MS signals, most of the compounds presented the *m*/*z* of the totally silylated molecule in their spectra, but MS signals corresponding to the loss of trimethylsilyl groups (-C_3_H_8_Si) as well as -OC_3_H_9_Si losses were also commonly found. Accordingly, considering X as the completely silylated molecule, [X + H]^+^, [X − C_3_H_8_Si + H]^+^, [X − C_6_H_16_Si_2_ + H]^+^, [X − C_9_H_24_Si_3_ + H]^+^, [X − C_12_H_32_Si_4_ + H]^+^ and [X − OC_3_H_9_Si + H]^+^ were defined as additionally possible ions (referred to as “common ions”) for features selection in MetaboScape. The rest of the extraction parameters, among which peak length and peak correlation stand out, were selected so as to have a reasonable number of putative compounds (approximately 2000) in each data matrix of the five resulting ones (one for each experiment: two interfaces, two polarities in LC-MS, and one in GC-MS; in other words, one for LC-ESI (+), one for LC-ESI (−), two for LC-APCI in positive and negative polarity, respectively, and one for GC-APCI in positive polarity).

**Figure 1:**
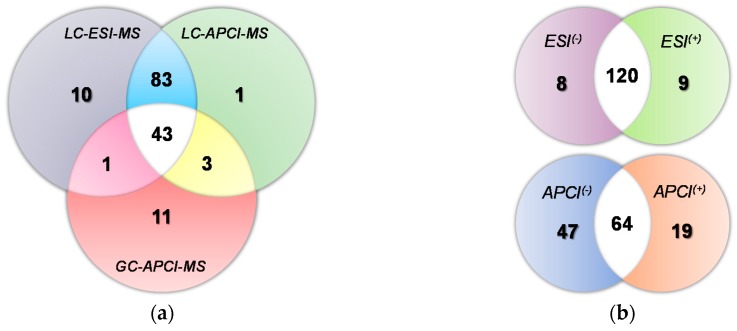
Venn diagrams showing total and overlapping numbers of identified compounds achieved with each platform and MS polarity. (**a**) LC-ESI-MS vs. LC-APCI-MS vs. GC-APCI-MS (combining together both ionization modes in LC-MS experiments); and (**b**) positive (+) vs. negative (−) polarity in LC-ESI-MS and LC-APCI-MS platforms.

Afterwards, all the available annotation strategies were applied in an attempt to give plausible identities to as many compounds as possible in the analyzed extracts. Figure 1a shows a comparison between the total number of annotated compounds accomplished by using the different platforms employed in this study (having into account both polarities to calculate the number corresponding to LC couplings). The GC-APCI-MS hyphenation gave the fewest number of putative compound identifications in the olive derived samples (58), although 11 of them could only be detected with this platform. With LC-MS methodologies, 137 and 130 compounds were identified using ESI and APCI sources, respectively; 126 being detected with both of them. As can be seen from Figure 1b, 129 was the highest number of compounds which could be identified in one run, specifically, operating the LC-ESI-MS platform in positive ionization mode. When using the negative mode for LC-ESI-MS analyses, only one compound less could be identified, 120 substances being correctly annotated by using both polarities. In the case of LC-APCI-MS analyses, negative ionization mode allowed the identification of 111 compounds whilst 83 were identified in positive polarity; 64 of them being detected in both polarities.

Table S1 shows the detected compounds in LC-MS using both ESI and APCI sources. It includes the assigned names, the calculated neutral molecular formulas (M), Rts and MS signals detected when using each interface in both positive and negative polarities. The presented *m*/*z*, error (difference between the observed mass and the theoretical one) and mSigma (goodness of fit between the measured and the calculated isotopic pattern) correspond to the major ion detected (appearing first in the row) in the “ESI MS signal” column (when available). The last column indicates if the compound identity was confirmed with the corresponding analytical standard or isolated fraction, if the identification was tentatively achieved with MetaboScape annotation tools (MetFrag or MS/MS library search), or if the peak assignment was done by contrasting previously published information about compounds already detected in olive-related matrices. A total of 141 annotated compounds, belonging to seven different chemical families, are presented in Table S1. 

**Organic acids.** The presence of quinic acid in the extracts was confirmed by analyzing the corresponding pure standard. In addition, the compound eluting at 0.7 min (calculated molecular formula C_6_H_8_O_7_), was tentatively annotated as citric acid (as previously reported in the literature [33]).

**Phenolic acids and aldehydes.** Five cinnamic acids (caffeic, *p*-coumaric, ferulic, sinapic, and *t*-cinnamic acids), eight benzoic acids (gallic, protocatechuic, gentisic, 4-hydroxybenzoic, 4-hydroxyphenylacetic, vanillic, syringic and homovanillic acids) and vanillin (a benzoic aldehyde) were annotated by matching with our in-house created analyte list. Moreover, 3,4,5-trimethoxybenzoic (known as eudesmic acid) and verbascoside (also known as acteoside), which is a hydroxycinnamic acid derivative, were tentatively identified in accordance with previous reports [34].

**Coumarins.** Two coumarins, aesculetin, and aesculin (aesculetin 6-*O*-glucoside) were found in the analyzed extracts. Suggested peaks agreed with relative Rts found by other authors [32,33].

**Simple phenols and derivatives.** The most popular substances of those found in olive matrices belonging to this family are hydroxytyrosol and tyrosol, which were unequivocally annotated by comparison with their analytical standards. Different derivatives of both of them (oxidized and acetylated hydroxytyrosol as well as hydroxytyrosol and tyrosol glucosides) were also found in some of the studied samples. Their identities were assigned having into account the changes of polarity caused by their distinctive functional groups and the way in which they theoretically should influence the eluting order. Another phenolic alcohol (3,4-dihydroxyphenylglycol) widely described in *Olea europaea* L. related matrices [34] and 2-phenethyl β-primeveroside, which has been previously isolated from olive cells [43], were also detected in the evaluated extracts. Besides, the peak with Rt 8.0 min and calculated molecular formula C_17_H_26_O_4_, was tentatively annotated as gingerol, a natural methoxyphenol which, as far as we know, has not been detected in olive tissues before. The outcome of MetFrag (conducted on LC-ESI-MS/MS (−) data) that helped to gingerol′s fragments assignment is represented in Figure S2a.

**Secoiridoids and derivatives.** This chemical class was represented by 49 compounds in total; the identity of 15 of them was confirmed with the corresponding pure standard or isolated fraction. Secoiridoids can occur in glycosidic or aglycone forms (as a result of enzymatic hydrolysis); a large number of intermediates and derived products can be found in olive tree derived matrices, resulting from their biosynthetic and degradation pathways [44]. One sub-group of secoiridoids included 20 compounds belonging to the oleuropein family (which presents hydroxytyrosol in their structure): oleuropein, hydroxyoleuropein, dihydrooleuropein (two isomers), oleuropein glucoside, oleuropein aglycone (six isomers), methyloleuropein aglycone, dimethyloleuropein aglycone, 10-hydroxyoleuropein aglycone (two isomers), dehydrooleuropein aglycone, decarboxymethyloleuropein aglycone (oleacein), hydroxydecarboxymethyloleuropein aglycone, methyldecarboxymethyloleuropein aglycone, and hydroxytyrosol acyclodihydroelenolate. Another sub-group corresponded to the nine homologous tyrosol derivatives (ligstroside family): ligstroside, ligstroside aglycone (six isomers), decarboxymethylligstroside aglycone (oleocanthal) and hydroxydecarboxymethylligstroside aglycone. The third sub-group was comprised of elenolic acid and 19 related compounds, including hydroxyelenolic acid (three isomers), desoxyelenolic acid (two isomers), decarboxymethylelenolic acid, hydroxydecarboxymethyl elenolic acid (two isomers), the decarboxylated form of hydroxyelenolic acid (two isomers), elenolic acid methylester, acyclodihydroelenolic acid hexoside, elenolic acid glucoside (also known as oleoside 11-methylester), oleoside or secologanoside (which are double-bond positional isomers), nuzhenide, comselogoside (two isomers), cafselogoside, and lucidumoside C.

**Flavonoids.** Flavonoids, which are widespread in plants and fruits, can have lots of structural variations that generate different sub-classes. In the analyzed extracts, eight flavonoids were found in aglycone form: a flavanone (naringenin), a flavanol (gallocatechin), a flavonol (quercetin), two flavanonols (dihydrokaempferol and taxifolin), and three flavones (luteolin, diosmetin and apigenin). MetFrag and MS/MS library search allowed the tentative annotation of C_15_H_12_O_6_ (3.6 min) as a kaempferol derivative, not previously detected in *Olea europaea* L. matrices (See Figure S2b). Eleven flavonoid glycosides were also identified in the evaluated samples. Three luteolin glucosides were detected at Rt 2.8, 3.1, and 3.2 min; the first one was identified as luteolin 7-*O*-glucoside (confirmed with the pure standard), the second one was annotated as luteolin 4′-*O*-glucoside (according to the Bruker Sumner MetaboBASE Plant Library) and the third one could be a different positional isomer or another kind of glycoside. The presence of rutin, quercetin 4′-*O*-glucoside, and apigenin 7-*O*-glucoside was confirmed with their pure standards too. A luteolin diglucoside isomer, cyanidin 3-*O*-glucoside, luteolin 7-*O*-rutinoside, apigenin 7-*O*-rutinoside and chrysoeriol 7-*O*-glucoside were also found in the evaluated olive tree derived samples. Tentative identification of positional isomers for those compounds which are not present in spectral libraries, was carried out on the basis of previously published reports [3,34].

**Lignans.** Pinoresinol, hydroxypinoresinol, acetoxypinoresinol, and syringaresinol, which have been widely described in olive oil and tissues, were also identified by LC-MS.

**Pentacyclic triterpenes.** Three triterpenic acids (maslinic, betulinic and oleanolic acids) and two triterpenic alcohols (erythrodiol and uvaol) were found in the extracts and unequivocally annotated thanks to our analyte list. Additionally, the peak eluting at 8.0 min (calculated molecular formula C_30_H_48_O_5_) was tentatively assigned to a maslinic acid monohydroxylated derivative, which has been described as a product of maslinic acid metabolism [45]. Its fragmentation pattern was characterized by a major signal corresponding to the dehydroxylated molecule and the decarboxylated maslinic acid moiety, which was also predominant in maslinic acid MS/MS spectrum.

**Tocopherols.** The four forms of tocopherols (α-, β-, γ-, and δ-) where found in some of the analyzed samples and annotated by comparison with their pure standard. Nevertheless, β- and γ-structural isomers could not be resolved in reverse-phase LC and coeluted in 12.7 min.

**Sterols.** Stigmasterol, campesterol, and β-sitosterol were annotated by matching with the in-house created analyte list. Two lupeol isomers, cycloartenol, stigmastadienol, Δ^5^-avenasterol, citrostadienol, and methylencycloartanol were also found in some of the prepared extracts; peak assignment was performed based on the occurrence and relative Rts described in previous reports [46,47,48].

**Fatty acids and derivatives.** Some of the most common fatty acids occurring in olive fruits and oils (stearic (C18:0), oleic (C18:1), linoleic (C18:2), linolenic (C18:3), palmitic (C16:0), and palmitoleic (C18:1) acids) were detected with the proposed LC-MS methodologies. Azelaic acid, which is a derived product from oleic acid oxidation, as well as different hydroxylated fatty acid derivatives (hydroxydecanoic, hydroxyoctadecatrienoic, hydroxyoctadecadienoic, hydroxyoctadecenoic, hydroxyoctadecanoic, hydroxyeicosanoic, dihydroxyhexadecanoic, dihydroxyoctadecanoic, dihydroxyoctadecadienoic, trihydroxyoctadecadienoic, trihydroxyoctadecenoic, and trihydroxyoctadecanoic acids), were also tentatively identified some of the evaluated samples. Those compounds have been reported as auto-oxidation products in heated edible fats [49], although some of them have been also found in olive leaves [50]. To the best of our knowledge, this is the first time that so many members of this family are found in olive derived matrices. 

Table S2 lists the 58 compounds detected with GC-APCI-MS. It includes names, M and Rts of the assigned peaks, as well as the qualitative information used for identification purposes: *m*/*z*, error, mSigma, calculated molecular formula and chemical arrangement corresponding to that formula, together with some other MS signals which helped to confirm the proposed identity (with their molecular formula between brackets). The most abundant *m*/*z* of each compound is presented in bold letters. 

As already mentioned, the number of compounds annotated using this platform was much lower than with the LC-MS couplings. On the one hand, all the glycosylated forms were undetectable by this methodology (under the selected conditions) and on the other hand, most secoiridoid derivatives presented a very similar in-source fragmentation that prevented the straightforward identification of all the individual molecules detected by LC-MS. Compound identification when using this GC-MS methodology was partially discussed in a previous report [37]; nevertheless, the use of a high resolution analyzer together with the APCI interface (which produced lower in-source fragmentation than the EI source used in the just mentioned publication) allowed the confirmation of some tentatively assigned identities.

Between those compounds exclusively detected with GC, we found squalene, a well-known hydrocarbon from VOO [37]; arachidic or eicosanoic acid (C20:0), whose hydroxylated derivative was tentatively identified with the LC-MS platforms; and glyceryl linoleate, which could come from triacyglycerols degradation. Additionally, additional isomers of apigenin, luteolin, maslinic acid, and elenolic acid (two isomers in this case) were detected in the analyzed extracts. In the case of both flavonoids, the detected isomers eluted earlier than the peak of the corresponding pure standard.

### 2.2. Comparison of the Potential of the Evaluated Analytical Platforms 

**Figure 2:**
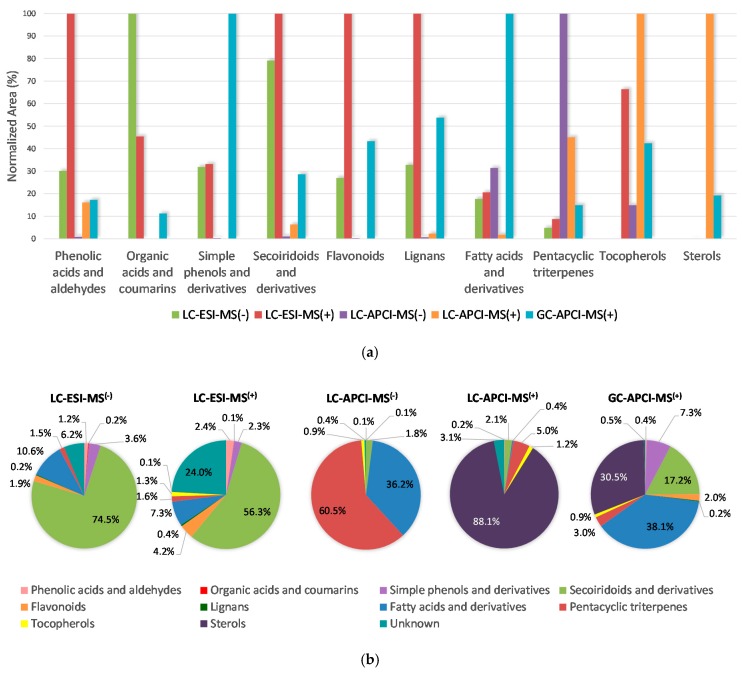
(**a**) Bars graph representing the sum of areas (in a normalized axis) of the compounds found in the oil obtained from stoned and dehydrated olives (grouped by chemical class), by means of each tested platform and polarity; (**b**) pie charts showing the share of every chemical class (in terms of area (% of the total area)) in the chromatograms obtained with each employed methodology for the same sample as in part (**a**).

One of the main objectives of the present work was to evaluate the adequacy of each tested methodology to determine different chemical classes of metabolites found in olive tree derived samples. Apart from the number of analytes which could be detected and tentatively annotated by using each platform and polarity (already discussed in Section 2.1 and clearly depicted in Figure 1), the efficiency of the ionization in each case was deeply evaluated. To illustrate this comparison, Figure 2 shows the efficiency of all the tested couplings when detecting different classes of compounds found in the oil produced from stoned and dehydrated olives. Two reasons made us selecting this sample to perform the comparison shown in the figure: (i) it was the matrix containing the second major number of total compounds (as it will be further described in Section 2.3), and (ii) it was the richest sample in terms of number of substances identified with the GC-MS platform. Bearing these two factors in mind, it could be considered as a very appropriate instance to illustrate the platforms comparison. In any case, similar charts and numerical comparisons were carried out for the rest of the matrices, corroborating the displayed observations regarding the efficiency of each platform to ionize every chemical family.

Figure 2a displays the normalized peak areas achieved for each chemical class with the five evaluated approaches (LC-ESI-MS (−/+), LC-APCI-MS (−/+) and GC-APCI-MS (+)). To facilitate the comparison, the highest area value (sum of all the compounds belonging to each group described in Section 2.1) was considered as 100, and the obtained areas with the rest of the tested platforms were expressed as a percentage of that value. It can be seen that the LC-ESI-MS platform, when working in positive ionization mode, produced the highest ionization rate for phenolic acids and aldehydes, secoiridoids and derivatives, flavonoids and lignans. The LC-ESI-MS methodology in negative polarity was the most appropriate one to detect the group of organic acids and coumarins, although it also showed relatively good efficiency when detecting secoiridoids and related substances. LC-ESI-MS (−) was also the second option to ionize phenolic acids and aldehydes with a suitable degree of effectiveness, and the third one (with very similar efficacy if compared with LC-ESI-MS (+)) for simple phenols and derivatives. The ESI interface (in any of both, positive or negative, polarities) was not useful for the determination of sterols. The LC-APCI-MS coupling used in negative ionization mode gave the best ionization rate for pentacyclic triterpenes (even though the alcohols were not ionizable in MS (−)), while, in positive polarity, it was the best option for tocopherols and sterols detection. As expected, LC-APCI conjunction resulted to be inadvisable for the detection of the most polar compounds. The GC-APCI-MS method was the best option for simple phenols and fatty acids-related analytes. It also gave good results for the rest of the considered chemical classes (in particular for lignans, tocopherols and sterols (if compared with the other approaches)), except for the previously mentioned fact that it was not possible to determine glycosylated compounds by means of this coupling (hence, the lower number of annotated metabolites in this platform). That means that the respective values shown in Figure 2 regarding the GC-APCI-MS platform do just consider aglycone forms. 

Pie charts presented in Figure 2b show the percentage (in terms of area) corresponding to each determined chemical class over the total area of the chromatograms obtained by means of the five methodologies used in this study. In view of the fact that some compounds remained as “unknown” (although we were able to assign them a molecular formula), we decided to include these substances in the systematic analytical comparison; doing it so we could have an idea about the percentage of the total area corresponding to non-identified substances in each platform (please, note that the analytes comprised in the unknown fraction are different in LC-MS than in GC-MS). Secoiridoids and derivatives group represented the highest area fraction of the chromatograms acquired with the ESI source in LC-MS, followed by fatty acids and derivatives, and the rest of phenolic compounds (simple phenols, flavonoids, phenolic acids and aldehydes and lignans) in different proportions depending on the selected MS polarity. The area corresponding to unknown peaks was also appreciable, accounting for 6% of the total area in negative polarity and for almost a quarter of the entire chromatogram in positive polarity. Pentacyclic triterpenes constituted around 1.5% of both (negative and positive) LC-ESI-MS chromatograms. As revealed in Figure 2a, the APCI interface in LC-MS produced better ionization for the less polar compounds. Therefore, in negative polarity, one third of the whole chromatogram area corresponded to fatty acids and derivatives, and almost the other two thirds were taken up by triterpenic acids. When using LC-APCI-MS (+), nearly 90% of the total area corresponded to sterols, 5% to pentacyclic triterpenes and around 3% to the unknown fraction. The chromatogram obtained by means of the GC-APCI-MS platform was more proportionally distributed. In this case, fatty acids and derivatives accounted for 38.1%, sterols for 30.5%, secoiridoids for 17.2%, simple phenols and derivatives for 7.3%, pentacyclic triterpenes for 3.0% and flavonoids for 2% of the chromatogram area. Logically, minor chemical classes such as organic acids, coumarins and lignans represented less than 1% regardless of the platform. It is also worth mentioning that tocopherols constituted around 1% of the total area obtained by all the evaluated methodologies, excluding LC-ESI-MS (−) (with which they were hardly detectable).

### 2.3. Establishing the Relative Prevalence of Each Determined Chemical Class in the Samples under Evaluation

**Figure 3:**
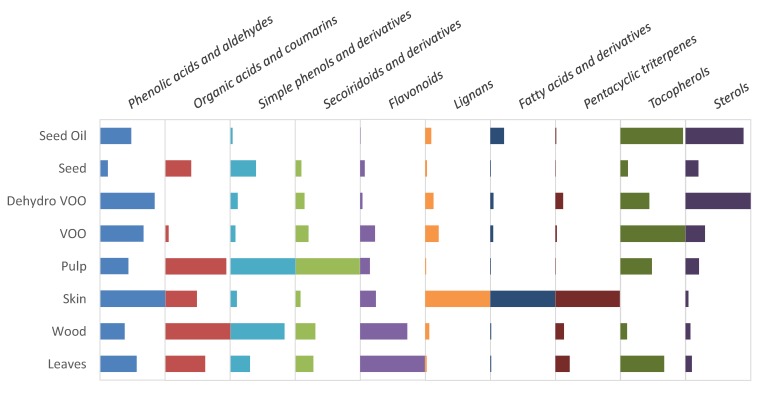
Bars graph representing relative distributions of each evaluated chemical class in the eight studied olive tree derived samples from Picudo cv.

Figure 3 presents the relative distribution of each determined chemical class in the evaluated samples. A similar strategy to the one described before was applied to facilitate the comparison. Thus, the integrated areas were normalized to the major value found for each family of compounds; in a subsequent step, the areas found in the rest of the matrices were expressed as a percentage of the richest one. As the peak intensity depends on the ionization rate of each individual substance, the followed strategy cannot be used to establish a comparison among different compound classes. The comparative purpose in this case, was consequently semi-quantitative and, as stated, just pertinent to collate the different samples considering each chemical class separately. Establishing absolute quantitative values of each analyte in every substance class was beyond the goal of this study. 

The distribution of the determined metabolites in the eight analyzed samples can be checked in Table S3 (all the given values are % referred to the richest sample regarding each analyte). In order to obtain comparable results among matrices, all the reported relative areas were integrated in chromatograms obtained by means of the same platform. Nevertheless, each chemical class was determined in the most favorable coupling (the one giving the maximum number of identified compound and good ionization rate avoiding saturation in any matrix): organic acids, coumarins and phenolic compounds (phenolic acids and aldehydes, simple phenols, secoiridoids, flavonoids, and lignans) in LC-ESI-MS (−); fatty acids and derivatives, as well as triterpenic acids in LC-APCI-MS (−); and triterpenic alcohols, tocopherols, and sterols in LC-APCI-MS (+).

Phenolic acids and derivatives were quite distributed over all the evaluated samples, fruit skin, and olive oils (obtained by any of the two procedures described in Section 3.2) being the richest matrices. The content of the oils in terms of organic acids was very low, probably because they are the most hydrophilic compounds among all the determined metabolites. Coumarins were almost exclusively found in stems; finding these substances in wood tissues is in good agreement with what was previously described by other authors for some olive tree varieties [31].

With regard to simple phenols, the glycosidic forms were mostly found in olive tissues, since they are generally hydrolyzed during oil extraction (for example, by the β-glucosidase action [8]). VOO was the richest matrix in terms of 3,4-dihydroxyphenylglycol. On the contrary, if compared with the oil obtained from stoned and dehydrated olives, the oil produced by the two-phase extraction method presented a reduced amount of the other two phenyl alcohols (tyrosol and hydroxytyrosol) and the acetylated derivative of hydroxytyrosol, but a higher content of the oxidized one. In addition, VOO was richer in terms of aglycones of oleuropein and ligstroside derivatives and had lower concentration of elenolic acid derivatives than the oil obtained from stoned and dehydrated olives; this can be seen in detail in Figure S3. It could suggest that either the thermal process involved in the dehydration of the stoned fruits is breaking down the secoiridoids into their degradation products (phenolic alcohols, elenolic acid, and derivatives) [8], or that the absence of water during the oil extraction is detrimental to the transfer of secoiridoids from the pulp to the oily phase. Figure S3 also shows how the glycosylated secoiridoids were more abundant in tissues than in the oils for the same reason as for the glycosylated simple phenols. Skin and seeds were the poorest olive tissues in terms of secoiridoids, being nuzhenide the most prevalent secoiridoid found in the latter one, as previously reported by different authors [51,52]. Glycosylated flavonoids were predominantly distributed between leaves and stems. The aglycones were also present in olive oils, more abundantly in VOO. Seeds and seed oil were the poorest matrices in terms of this chemical class, but they contained noticeable amounts of lignans, seed oil being the richest matrix regarding acetoxypinoresinol. Olive fruit skin was the matrix with the highest content of the other three evaluated lignans (syringaresinol, pinoresinol, and hydroxypinoresinol).

Although fatty acids are usually found as part of triacylglycerols, they were detected free in the three kind of oils evaluated in this study. Moreover, the compounds tentatively annotated as fatty acid hydroxylated derivatives, were found in high relative amounts in olive skin. As far as triterpenic compounds are concerned, olive skin was the richest matrix, followed by leaves, except for betulinic acid that was found at a higher relative concentration level in the stems. The oil obtained from stoned and dehydrated olives presented higher relative triterpenoids content than the VOO obtained from the conventional procedure. Additionally, those compounds were found at very low relative levels in olive seed and pulp (what is in agreement with previous findings [53]). Regarding tocopherols, VOO was the matrix containing the highest relative amount of α-tocopherol, while δ-, β-, and γ-tocopherols were higher, in relative terms, in the seed oil and the oil obtained from stoned and dehydrated olives. The latter was the richest matrix in terms of sterols, followed by VOO (considering the overall distribution of all the determined sterols). Just campesterol, citrostadienol, and β-sitosterol were found at higher relative levels in seed oil. Some sterols were found at low relative concentrations in pulp and seeds; they were almost missing from the rest of olive tree derived tissues (leaves, stems, and olive skin).

## 3. Materials and Methods 

### 3.1. Chemicals and Standards

Deionized water produced by a Millipore Milli-Q system (Bedford, MA, USA) and acetonitrile of LC-MS grade supplied from Sigma-Aldrich (St. Louis, MO, USA), were acidified with 0.5% acetic acid (provided by Sigma-Aldrich too), and used as mobile phases in LC. Gradient grade ethanol for sample preparation was purchased from Merck (Madrid, Spain). *N*,*O*-bis(trimethylsilyl)trifluoroacetamide with 1% of trimethylchlorosilane, (BSTFA + 1% TMCS) used as derivatization reagent and 43 pure standards of metabolites found in *Olea europaea* matrices were acquired from Sigma-Aldrich. The list of standard compounds included: phenolic compounds (hydroxytyrosol, tyrosol, oleuropein, luteolin, luteolin 7-*O*-glucoside, apigenin, apigenin 7-*O*-glucoside, quercetin, quercetin 4-*O*-glucoside, rutin, pinoresinol, vanillin and quinic, gallic, protocatechuic, gentisic, 4-hydroxybenzoic, 4-hydroxyphenylacetic, vanillic, caffeic, syringic, homovanillic, *p*-coumaric, sinapic, ferulic, and *t*-cinnamic acids); triterpenic compounds (maslinic, betulinic, oleanolic and ursolic acids, erythrodiol and uvaol); fatty acids (palmitoleic, oleic, linoleic and linolenic acids); tocopherols (α-, β-, γ-, and δ-tocopherols), and sterols (stigmasterol, campesterol, and β-sitosterol). Additionally, isolated fractions of secoiridoids (oleuropein and ligstroside aglycones, oleacein, and oleocanthal), elenolic acid, acetylated hydroxytyrosol, and acetoxypinoresinol, which were not commercially available, were also used for identification purposes.

### 3.2. Samples and Sample Treatment

**Figure 4:**
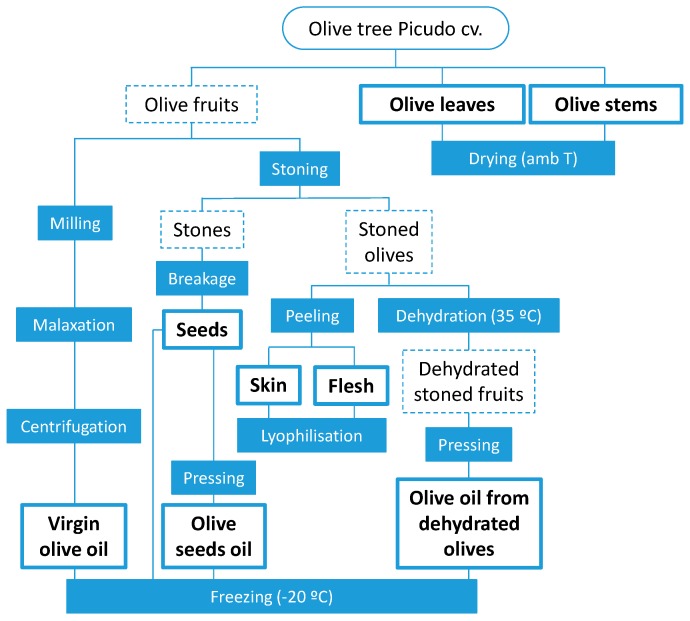
Diagram of the procedure followed to obtain the 8 samples studied in this work, including intermediate products (dotted lines) and employed processes (shaded boxes).

Sampling of olive fruits, leaves and stems, was performed on the same Picudo cv. olive tree mucho grown in Castillo de Locubín (Jaén, Spain (at approximately 750 m above sea level)) in January 2017. The olive tree cultivar was declared by the producer and had been previously certified. In total, eight different samples (tissues and oils) were analyzed in this study. Leaves and stems were dried at ambient temperature and stored in a fresh dark place. A portion of 5 kg of fresh fruits was processed by means of an Abencor^®^ laboratory oil mill (MC2 Ingeniería y Sistemas, Seville, Spain) to obtain VOO at laboratory scale by the conventional two-phase process, which involves three steps: (i) crushing of entire fruits, (ii) malaxation of the paste and (iii) centrifugation for oil separation. Official IOC determinations were carried out to confirm the belonging of the oil to VOO category. The rest of the fruits were manually deconstructed to obtain different tissues and oils. Firstly, fruits were stoned and the obtained pits were broken with a hammer to extract the olive seed contained inside. Next, half of the stoned fruits were peeled to obtain olive skin and pulp separately, which were straightaway frozen and freeze-dried. The other half of the stoned fruits were dried at 50 °C in an oven until constant weight. Olive seeds and dehydrated stoned fruits were further processed by mechanical pressing to obtain two new kinds of oils. Figure 4 shows a diagram of the procedure followed to prepare the samples.

Both kinds of samples (tissues and oils) were subjected to a very unselective sample treatment, trying to extract compounds belonging to different chemical classes in a wide range of polarity. Thus, two ethanol/water mixtures were applied in a liquid-liquid or solid-liquid extraction protocol adapted from that previously suggested by our research team [37,54]. For liquid samples, 1 g of oil was extracted three times with 6 mL ethanol/water mixtures, whereas for solid samples, 0.5 g of the grinded and sieved tissue were extracted three times with 10 mL of the extractant agent. In both cases, the first extraction step was done with ethanol/water (60:40, *v*/*v*), while for the last two steps, ethanol/water (80:20, *v*/*v*) was used instead. Olive tissues extraction was carried out in an ultrasonic bath from J.P. Selecta (Barcelona, Spain) for 30 min whilst 4 min of vortex shaking where enough to mix the phases in oily samples and to assure an efficient extraction. After collecting together the supernatants from the three extraction steps, the solvent was evaporated in a rotavap and the residue was reconstituted in the appropriate volume of ethanol/water (80:20, *v*/*v*) (1 mL for the oils and 5 mL for the olive tissues). For GC analyses, a 50 μL aliquot of the prepared extracts was dried and then derivatized with 75 μL of BSTFA + 1% TMCS (keeping it at ambient temperature for 1 h) before injection into the chromatograph, following a previously reported strategy [37,54].

### 3.3. GC-MS and LC-MS Methodologies

GC-MS analyses were carried out in a Bruker 450-GC (Bruker Daltonik GmbH, Bremen, Germany) coupled to a Compact^TM^ QqTOF mass spectrometer (Bruker Daltonik) through an APCI source. 1 μL of the silylated extract was injected at a split ratio of 1:20 with an injector temperature of 250 °C. Analytes were separated in a BR-5 column (30 m × 0.25 mm i.d., 0.25 μm) (Bruker Daltonik) with 1 mL/min of He as carrier gas and a linear temperature gradient from 150 to 320 °C at a rate of 4 °C/min. The experimental conditions of the GC-MS method were described elsewhere to determine minor components of VOO [54]. 

LC-MS analyses were performed in an Elute UHPLC (Bruker Daltonik) coupled to the same MS detector as in GC-MS. Two different interfaces were used in this case, APCI and ESI. Analytes were eluted slightly modifying the previously published conditions [54], in an Intensity Solo C18 column (2.1 × 100 mm, 1.8 μm) (Bruker Daltonik), using acidified water (phase A) and ACN (phase B) with the following gradient: 0 to 2 min, 5–30% B; 2 to 7 min, 30–50% B; 7 to 8 min, 50–90% B; 8 to 8.2 min, 90–95% B, 8.2 to 10 min, 95–99.9% B (kept for 5.9 min), and 15.9 to 16 min, 99.9–5% B (kept for two post-run min). The flow rate was 0.4 mL/min from 0 to 10 min, and 0.6 mL/min from 10 min to the end of the run. The already reported detection conditions for ESI-QqTOF MS [54] were also used in this study. When the LC-MS coupling was done through the APCI interface, nebulizer pressure was set at 2 bars; drying gas flow and temperature were set at 2 L/min and 300 °C, apiece; capillary voltages were set at 2500 V in negative polarity and 2000 V in positive one; and 6000 and 10,000 nA were chosen for corona current in negative and positive ionization modes, respectively. Auto MS/MS fragmentation was also carried out in LC-MS analyses in order to facilitate compound identification. An absolute threshold of 1500 counts and a cycle time of 1 s were selected for precursor ions collection; collision energy stepping factors fluctuated between 0.2% and 0.8%.

GC-MS analyses were internally calibrated by comparison with known *m*/*z* from common cyclic-siloxanes found in the background. In LC-MS, an external calibrant was directly pumped into the interface at the beginning of each run using a Cole Parmer syringe pump (Vernon Hills, IL, USA), equipped with a Hamilton syringe (Reno, NV, USA). The calibrant for LC-ESI-MS analyses consisted in a mix of clusters of sodium formate and acetate, while the one used in LC-APCI MS was a mixture of analytical standards (available in our lab) including an APCI tuning mix and six pesticides of known *m*/*z* in the range from 121 to 955.

Data acquisition was done with the software Compass HyStar and data treatment with DataAnalysis 4.4 and MetaboScape^®^ 3.0 (the three of them from Bruker Daltonik). The latter one automatically recalibrated the acquired MS data and performed the molecular features selection, bucketing, filtering and scaling. MetaboScape incorporates different tools that helped to identify the compounds found in the chromatograms: SmartFormula, which determines the molecular formula of each detected compound from its exact mass and isotopic pattern (having into account all found adducts); Compound Crawler, which searches molecular structures for given molecular formulas in local (AnalyteDB) and public online databases (ChEBI, ChemSpider, and PubChem); and MetFrag, which performs in silico fragmentation of the potential structures and compares them with the acquired MS/MS spectra [55,56]. This software also allows annotation by comparison with previously created analyte lists and MS/MS spectral libraries (Bruker Sumner MetaboBASE Plant Libraries 1.0 and Bruker HMDB Metabolite Library).

## 4. Conclusions

Eight interesting matrices coming from a Picudo cv. olive tree have been analyzed by powerful LC-ESI/APCI-QTOF MS and GC-APCI-QTOF MS methodologies, providing a comprehensive coverage of their secondary metabolites (141 substances identified in LC-MS and 58 in GC-MS) and giving reliable information about their phytochemical distribution. The suitability of each platform and polarity to determine each family of metabolites was systematically evaluated. When the selected matrices were compared by using a semi-quantitative approach, fruit skin resulted to be the matrix with the highest relative amounts of phenolic acids, triterpenic and fatty acid hydroxylated substances, exhibiting remarkable relative content of lignans too. Coumarins were almost exclusively found in stems. The glycosidic simple phenols and glycosylated secoiridoids were more abundant in tissues, as well as the glycosylated flavonoids (predominantly distributed between leaves and stems). VOO was the matrix showing highest relative content of 3,4-dihydroxyphenylglycol, aglycones of oleuropein and ligstroside derivatives, flavonoids (aglycones) and α-tocopherol. The oil obtained from stoned and dehydrated olives (in comparison with VOO) had relatively raised levels of tyrosol, hydroxytyrosol, the acetylated derivative of hydroxytyrosol, sterols and elenolic acid derivatives. Seed oil stood out for its notable levels of acetoxypinoresinol and tocopherols.

## Supplementary Materials

The following are available online. Figure S1: Extracted ion chromatograms (EICs) of all the identified compounds in olive oil obtained from stoned and dehydrated fruits, when it is analyzed by means of each evaluated platform and polarity; Figure S2: MetFrag in silico fragmentation for two tentatively annotated metabolites (A and B), and spectral library match for compound B; Figure S3: Distribution of secoiridoids in the eight matrices under study (representation of the sum of absolute areas); Table S1: List of compounds detected with LC-MS methodologies; Table S2: List of compounds identified with GC-APCI-MS; Table S3: Distribution of the determined metabolites in the eight evaluated samples (all the given values are percentages referred to the richest sample regarding each analyte).
