# Supplementary material for: Unravelling the Distribution of Secondary Metabolites in Olea europaea L.: Exhaustive Characterization of Eight Olive-Tree Derived Matrices by Complementary Platforms (LC-ESI/APCI-MS and GC-APCI-MS)
Source: Molecules. 2018 Sep 20;23(10):2419. doi: 10.3390/molecules23102419 (PMC6222318; doi:10.3390/molecules23102419)
Supplement: Supplementary file 1 [file molecules-23-02419-s001.pdf]

SUPPLEMENTARY MATERIAL

Unravelling the Biochemical Distribution of Secondary Metabolites in *Olea Europaea* L.: Exhaustive Characterization of Eight Olive-Tree Derived Matrices by Complementary Platforms (LC-ESI/APCI-MS and GC-APCI-MS)

Lucía Olmo-García[1], Nikolas Kessler[2], Heiko Neuweger[2], Karin Wendt[2], Alberto Fernández-Gutiérrez[1], José María Olmo-Peinado[3], Carsten Baessmann[2], Alegría Carrasco-Pancorbo[1*]

[1] Department of Analytical Chemistry, Faculty of Science, University of Granada, Ave. Fuentenueva s/n, 18071, Granada, Spain.
[2] Bruker Daltonik GmbH, Fahrenheitstraße 4, 28359 Bremen, Germany.
[3] Acer Campestres S.L., Almendro, 37 (Pol. Ind. El Cerezo), 23670, Castillo de Locubín, Jaén, Spain

[*]Corresponding author: alegriac@ugr.es

Index

Figure S1. Extracted Ion Chromatograms (EICs) of all the identified compounds in olive oil obtained from stoned and dehydrated fruits, when it is analyzed by means of each evaluated platform and polarity.

Figure S2. MetFrag *in-silico* fragmentation for two tentatively annotated metabolites (A and B), and spectral library match for compound B.

Figure S3. Distribution of secoiridioids in the eight matrices under study (representation of the sum of absolute areas).

Table S1. List of compounds detected with LC-MS methodologies.

Table S2. List of compounds detected with GC-APCI-MS.

Table S3. Distribution of the determined metabolites in the eight evaluated samples (all the given values are % referred to the richest sample regarding each analyte).

Figure S1. Extracted Ion Chromatograms (EICs) of all the identified compounds in olive oil obtained from stoned and dehydrated fruits, when it is analyzed by means of each evaluated platform and polarity.

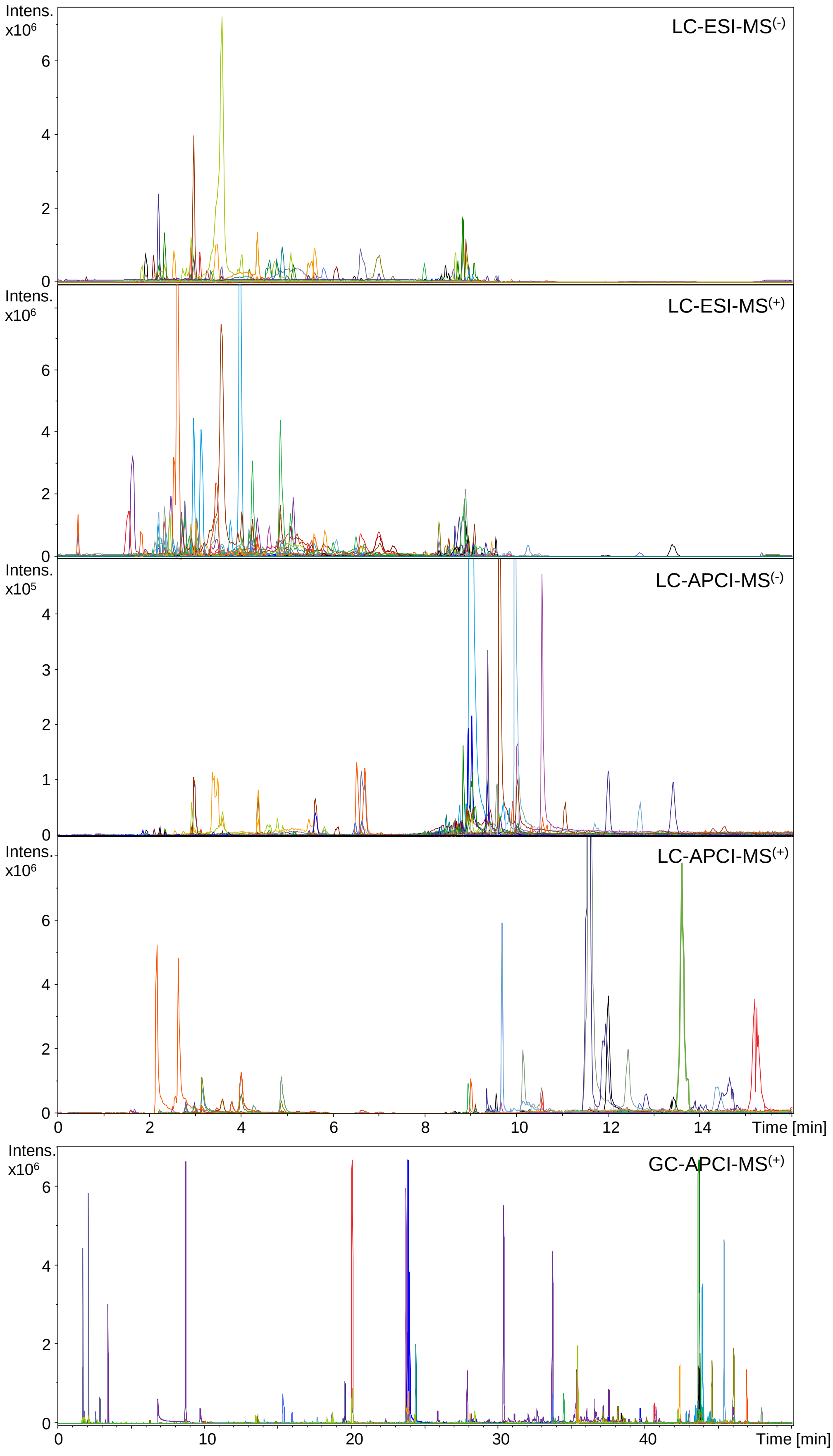

Figure S2. MetFrag *in-silico* fragmentation for two tentatively annotated metabolites (A and B), and spectral library match for compound B.

A) Gingerol

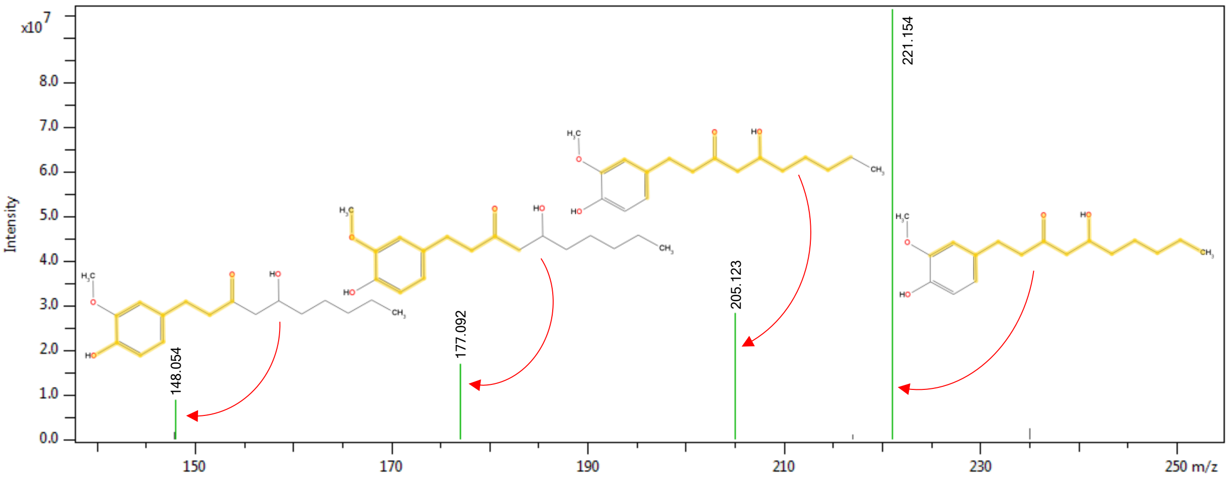

B) Dihydrokaempferol

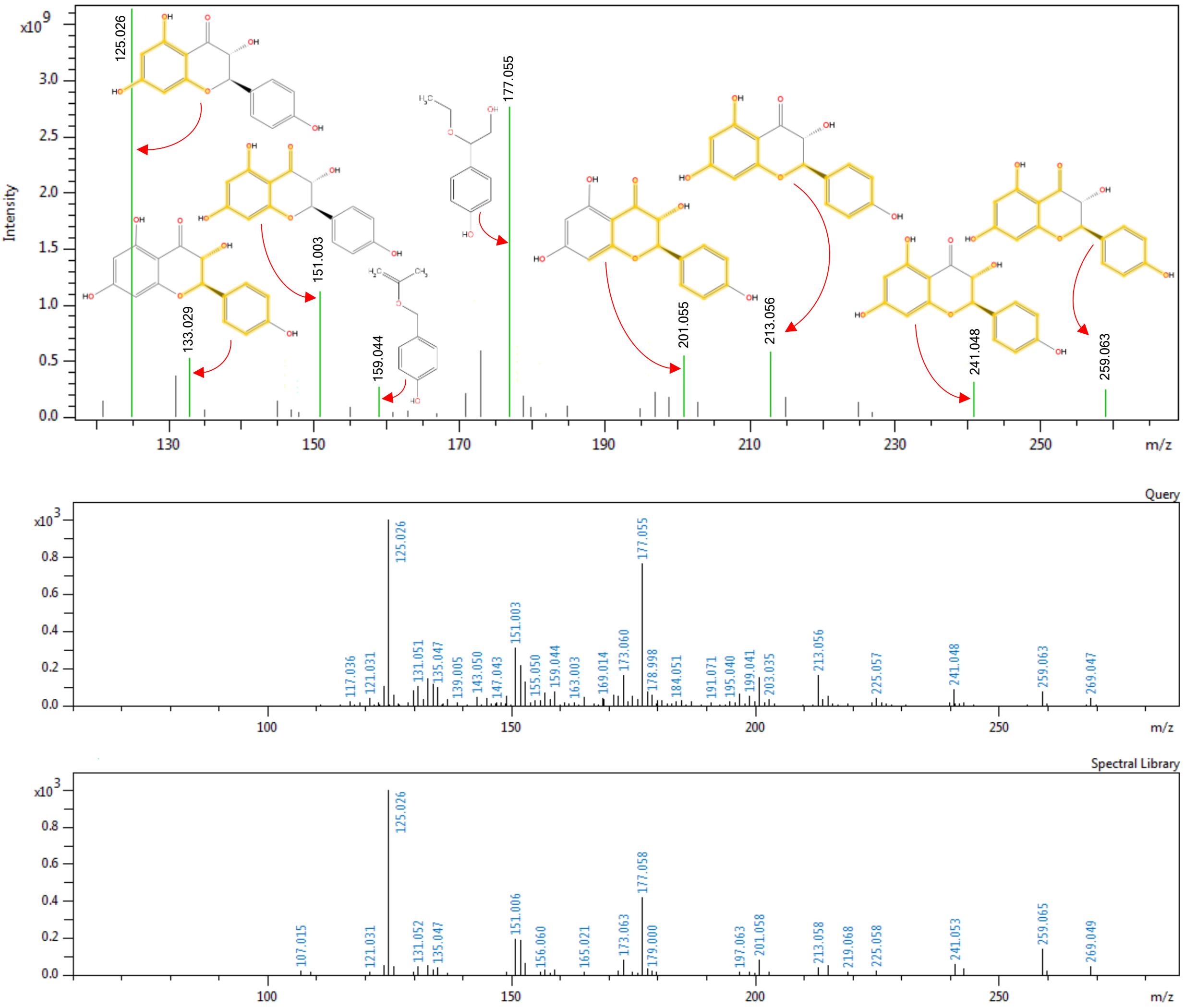

Figure S3. Distribution of secoiridoids in the eight matrices under study (representation of the sum of absolute peak areas).

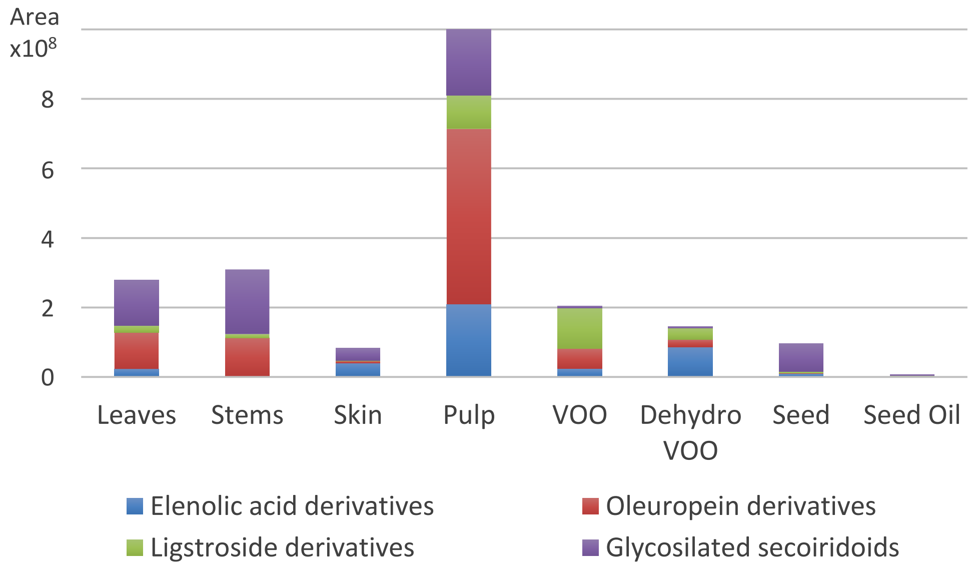

Table 1. List of compounds detected with LC-MS methodologies.

Compound	Neutral Molecular formula	Rt (min)	Negative					Positive					ID
			m/z	Error (mDa)	mSigma	ESI MS signal	APCI MS signal	*m/z*	Error (mDa)	mSigma	ESI MS signal	APCI MS signal	
quinic acid	$C_7H_{12}O_6$	0.6	191.0555	-0.621	8.8	$[M-H]^-$	-	193.0710	0.318	8.7	$[M+H]^+$, $[M-H_2O+H]^+$	$[M+H]^+$	S
citric acid	$C_6H_8O_7$	0.7	191.0191	-0.651	3	$[M-H]^-$	-	175.0238	0.052	8.4	$[M-H_2O+H]^+$, $[M+H]^+$	-	L
3,4-dihydroxyphenylglycol	$C_8H_{10}O_4$	0.8	151.0399	-0.342	8.9	$[M-H_2O-H]^-$, $[M-H]^-$	-	153.0544	-0.235	20.1	$[M-H_2O+H]^+$, $[M+H]^+$	$[M+H]^+$	L
oxydized hydroxytyrosol	$C_8H_8O_3$	1.0	151.0398	-0.808	15.6	$[M-H]^-$	$[M-H]^-$	153.0536	0.092	8.9	$[M+H]^+$, $[M-H_2O+H]^+$	-	L
hydroxytyrosol glucoside	$C_{14}H_{20}O_8$	1.7	315.1085	-0.065	3.8	$[M-H]^-$	$[M-H]^-$	317.1235	0.357	5.7	$[M+H]^+$, $[M-H_2O+H]^+$	$[M+H]^+$, $[M-H_2O+H]^+$	L
hydroxydecarboxymethylelenolic acid isomers I and II	$C_9H_{12}O_5$	1.7, 2.1	199.0607	-0.473	8.5	$[M-H]^-$	$[M-H]^-$	183.0654	0.230	9	$[M-H_2O+H]^+$, $[M+H]^+$, $[M+Na]^+$	-	L
gallic acid	$C_7H_6O_5$	1.8	169.0140	-0.793	26.6	$[M-H]^-$	-	-	-	-	-	-	S
acyclodihydroelenolic acid hexoside	$C_{17}H_{28}O_{11}$	1.8	407.1560	0.105	11.1	$[M-H]^-$	$[M-H]^-$	409.1701	-0.476	6.3	[M+H]+, [M-H2O+H]+, [M+Na]+	$[M+H]^+$	L
decarboxylated form of hydroxyelenolic acid isomers I and II	$C_{10}H_{14}O_5$	1.9, 2.2	213.0768	-0.044	9.2	$[M-H]^-$, $[M-H_2O-H]^-$	[M-H]-, $[M-H-H_2O]^-$	197.0813	0.614	15.2	$[M-H_2O+H]^+$, $[M+H]^+$, $[M+Na]^+$	$[M-H_2O+H]^+$, $[M+H]^+$	L
hydroxytyrosol	$C_8H_{10}O_3$	1.9	153.0551	-0.594	0.2	$[M-H]^-$	$[M-H]^-$	155.0700	-0.276	7.7	$[M+H]^+$	$[M+H]^+$	S
protocatechuic acid	$C_7H_6O_4$	1.9	153.0190	-0.309	8.2	$[M-H]^-$	$[M-H]^-$	155.0338	-0.644	12.5	$[M+H]^+$, $[M-H_2O+H]^+$	-	S
tyrosol glucoside	$C_{14}H_{20}O_7$	2.0	299.1139	0.191	1.6	$[M-H]^-$	$[M-H]^-$	301.1292	0.870	19.2	$[M+H]^+$	-	L
aesculin	$C_{15}H_{16}O_9$	2.0	339.0720	0.026	17.2	$[M-H]^-$	-	341.0877	1.033	0.4	$[M+H]^+$	$[M+H]^+$	L
dihydrooleuropein	$C_{25}H_{36}O_{13}$	2, 2.9	543.2082	-1.109	28.6	$[M-H]^-$, $[M-H_2O-H]^-$	[M-H]-, $[M-H-H_2O]^-$	-	-	-	-	-	L
oleoside/secologanoside	$C_{16}H_{22}O_{11}$	2.1	389.1091	0.014	4.7	$[M-H]^-$	$[M-H]^-$	391.1165	0.163	7.6	$[M+H]^+$	-	L
eudesmic acid	$C_{10}H_{12}O_5$	2.2	211.0607	0.236	28.9	$[M-H]^-$	$[M-H]^-$	231.0766	0.092	3.5	$[M+H]^+$	-	L
tyrosol	$C_8H_{10}O_2$	2.3	137.0608	-0.006	6.2	$[M-H]^-$	$[M-H]^-$	121.0642	-0.628	4.6	$[M-H_2O+H]^+$, $[M+H]^+$, $[M+Na]^+$	-	S
gentisic acid	$C_7H_6O_4$	2.3	153.0119	-0.309	8.2	$[M-H]^-$	-	155.0338	-0.644	20.1	$[M+H]^+$	-	S
luteolin diglucoside	$C_{27}H_{30}O_{16}$	2.3	609.1461	1.746	30.1	$[M-H]^-$	-	611.1608	-0.409	20.1	$[M+H]^+$	-	L
cyanidin 3-O-glucoside	$C_{21}H_{22}O_{11}$	2.3	449.1095	0.530	3.4	$[M-H]^-$	$[M-H]^-$	451.1217	-0.755	17.3	$[M+H]^+$	-	L
4-hydroxybenzoic acid	$C_7H_6O_3$	2.4	137.0246	0.339	8.2	$[M-H]^-$	-	139.0384	-0.512	5.5	$[M+H]^+$	$[M+H]^+$	S
elenolic acid glucoside	$C_{17}H_{24}O_{11}$	2.4	403.1246	0.093	9.3	$[M-H]^-$	$[M-H]^-$	405.1396	-0.383	21.2	$[M+H]^+$, $[M-H_2O+H]^+$	$[M+H]^+$	L
4-hydroxyphenylacetic acid	$C_8H_8O_3$	2.4	151.0397	-0.279	5.6	$[M-H]^-$	$[M-H]^-$	153.0542	-0.133	7.2	$[M+H]^+$	-	S
unknown 1	$C_{16}H_{26}O_{10}$	2.4	377.1453	0.000	5.7	$[M-H]^-$	$[M-H]^-$	379.1604	0.500	23.3	$[M+H]^+$	-	-
aesculetin	$C_9H_6O_4$	2.5	177.0188	-0.923	3.3	$[M-H]^-$	$[M-H]^-$	179.0340	-0.163	27.6	$[M+H]^+$	$[M+H]^+$	L
vanillic acid	$C_8H_8O_4$	2.5	167.0345	-0.459	7.1	$[M-H]^-$	$[M-H]^-$	169.0497	-0.868	30.6	$[M+H]^+$	-	S
caffeic acid	$C_9H_8O_4$	2.5	179.0347	-0.310	8.3	$[M-H]^-$	-	181.0495	-0.036	29.1	$[M+H]^+$, $[M-H_2O+H]^+$	$[M+H]^+$	S
gallocatechin	$C_{15}H_{14}O_7$	2.6	305.0702	3.507	27.6	$[M-H]^-$	-	-	-	-	-	-	L
unknown 2	$C_9H_{14}O_3$	2.6	169.0869	0.135	17.1	$[M-H]^-$	-	171.1016	0.012	6.2	$[M+H]^+$, $[M-H_2O+H]^+$, $[M+Na]^+$	$[M+H]^+$, $[M-H_2O+H]^+$	-
decarboxymethylelenolic acid	$C_9H_{12}O_4$	2.6	183.0658	-0.526	8	$[M-H]^-$	[M-H]-, $[M-H-H_2O]^-$	185.0812	0.348	7.8	$[M+H]^+$, $[M-H_2O+H]^+$	$[M+H]^+$	L

Compound	Neutral Molecular formula	Rt (min)	Negative					Positive					ID
			m/z	Error (mDa)	mSigma	ESI MS signal	APCI MS signal	*m/z*	Error (mDa)	mSigma	ESI MS signal	APCI MS signal	
2-phenethyl *β*-primeveroside	$C_{19}H_{28}O_{10}$	2.6	415.1612	0.102	3.8	$[M-H]^-$	$[M-H]^-$	417.1749	-2.830	41.3	$[M+H]^+$	$[M+H]^+$	L
unknown 3	$C_{18}H_{34}O_{14}$	2.6	473.1863	1.278	40.3	$[M-H]^-$	$[M-H]^-$	475.2054	0.976	30.2	$[M+H]^+$	-	-
syringic acid	$C_9H_{10}O_5$	2.6	197.0452	-0.004	12.4	$[M-H]^-$	$[M-H]^-$	199.0601	-0.402	8.9	$[M+H]^+$	-	S
homovanillic acid	$C_9H_{10}O_4$	2.7	181.0502	-0.778	13.6	$[M-H]^-$	$[M-H]^-$	183.0655	0.051	36.9	$[M+H]^+$	$[M+H]^+$	S
rutin	$C_{27}H_{30}O_{16}$	2.7	609.1464	0.278	5	$[M-H]^-$	$[M-H]^-$	611.1615	0.290	25.9	$[M+H]^+$	-	S
luteolin rutinoside	$C_{27}H_{30}O_{15}$	2.7	593.1516	0.047	27.3	$[M-H]^-$	$[M-H]^-$	595.1671	0.805	26.2	$[M+H]^+$	-	L
hydroxyoleuropein	$C_{25}H_{32}O_{14}$	2.7	555.1717	-0.391	4.7	$[M-H]^-$	[M-H]-, $[M-H-H_2O]^-$	-	-	-	-	-	L
p-coumaric acid	$C_9H_8O_3$	2.8	163.0397	-0.107	11.8	$[M-H]^-$	$[M-H]^-$	165.0545	-0.084	7.3	$[M+H]^+$, $[M-H_2O+H]^+$, $[M+Na]^+$	$[M+H]^+$	S
quercetin 4-O-glucoside	$C_{21}H_{20}O_{12}$	2.8	463.0882	-0.010	29.3	$[M-H]^-$	$[M-H]^-$	465.1007	-2.035	30.8	$[M+H]^+$	$[M+H]^+$	S
luteolin 7-O-glucoside	$C_{21}H_{20}O_{11}$	2.8	447.0933	0.009	11	$[M-H]^-$	$[M-H]^-$	449.1078	-0.586	17.2	$[M+H]^+$	$[M+H]^+$	S
verbascoside	$C_{29}H_{36}O_{15}$	2.8	623.1981	-0.003	14.6	$[M-H]^-$	-	625.2139	-0.840	31.7	$[M+H]^+$	-	L
oleuropein glucoside	$C_{31}H_{42}O_{18}$	2.9	701.2303	0.503	7.7	$[M-H]^-$	$[M-H]^-$	-	-	-	-	-	L
apigenin 7-O-rutinoside	$C_{27}H_{30}O_{14}$	2.9	577.1568	1.094	29.5	$[M-H]^-$	-	579.1701	-0.732	18.6	$[M+H]^+$	$[M+H]^+$	SL
hydroxyelenolic acid isomers I, II and III	$C_{11}H_{14}O_7$	2.9, 3.1, 3.3	257.0669	0.187	12.4	$[M-H]^-$, $[M-H_2O-H]^-$	$[M-H-H_2O]$-, $[M-H]^-$	259.0816	1.412	12.9	$[M+H]^+$, $[M-H_2O+H]^+$	$[M+H]^+$	L
nuzhenide	$C_{31}H_{42}O_{17}$	2.9	685.2346	-0.005	5.8	$[M-H]^-$	$[M-H]^-$	507.1867	0.600	12.9	$[M-C_6H_{12}O_6+H]^+$	-	L
sinapic acid	$C_{11}H_{12}O_5$	2.9	223.0615	-0.045	9.9	$[M-H]^-$	$[M-H]^-$	225.0765	0.482	11.8	$[M+H]^+$	$[M+H]^+$, $[M-H_2O+H]^+$	S
cafselogoside	$C_{25}H_{28}O_{14}$	2.9	551.1409	0.307	8	$[M-H]^-$, $[M-H_2O-H]^-$	-	-	-	-	-	-	L
unknown 4	$C_{11}H_{16}O_6$	3.0	243.0876	0.141	11.1	$[M-H]^-$	$[M-H]^-$	245.1025	0.486	13.7	$[M+H]^+$, $[M+Na]^+$, $[M+K]^+$	$[M-H_2O+H]^+$, $[M+H]^+$	-
vanillin	$C_8H_8O_3$	3.0	151.0396	-1.103	9.8	$[M-H]^-$	$[M-H]^-$	153.0543	0.567	14.1	$[M+H]^+$	$[M+H]^+$	S
ferulic acid	$C_{10}H_{10}O_4$	3.1	193.0503	-0.570	7.6	$[M-H]^-$	$[M-H]^-$	195.0658	0.597	9.6	$[M+H]^+$, $[M-H_2O+H]^+$	$[M-H_2O+H]^+$, $[M+H]^+$	S
luteolin 4'-O-glucoside	$C_{21}H_{20}O_{11}$	3.1	447.0934	0.075	13.4	$[M-H]^-$	$[M-H]^-$	449.1057	-0.638	16.2	$[M+H]^+$	$[M+H]^+$	SL
taxifolin	$C_{15}H_{12}O_7$	3.1	303.0509	0.079	18.3	$[M-H]^-$	[M-H]-, $[M-H-H_2O]^-$	305.0661	0.206	27	$[M+H]^+$, $[M-H_2O+H]^+$	$[M+H]^+$	L
apigenin 7-O-glucoside	$C_{21}H_{20}O_{10}$	3.1	431.0990	0.667	26.9	$[M-H]^-$	$[M-H]^-$	433.1124	-0.506	5.5	$[M+H]^+$	$[M+H]^+$	S
quercetin glucoside isomer	$C_{21}H_{20}O_{12}$	3.1	463.0884	-0.068	6.1	$[M-H]^-$	-	465.1001	-2.035	30.8	$[M+H]^+$	-	L
desoxyelenolic acid	$C_{11}H_{14}O_5$	3.1, 3.6	225.0771	-0.292	7	$[M-H]^-$	$[M-H]^-$	227.0913	-0.415	21.3	$[M+H]^+$	[M+H]+, $[M-H_2O+H]^+$	L
luteolin glucoside isomer	$C_{21}H_{20}O_{11}$	3.2	447.0914	-0.259	11	$[M-H]^-$	$[M-H]^-$	449.1056	-0.749	9.4	[M+H]+	$[M+H]^+$	L
chrysoeriol 7-O-glucoside	$C_{22}H_{22}O_{11}$	3.2	461.1078	-0.885	31.8	$[M-H]^-$	$[M-H]^-$	463.1235	-0.537	20.3	$[M+H]^+$	$[M+H]^+$	SL
comselogoside isomers I and II	$C_{25}H_{28}O_{13}$	3.2, 3.6	535.1457	0.014	37.5	$[M-H]^-$	[M-H]-, $[M-H-H_2O]^-$	537.1527	-3.132	39.1	$[M+H]^+$	$[M+H]^+$	L
10-hydroxyoleuropein aglycone isomers I and II	$C_{19}H_{22}O_9$	3.3, 4.1	393.1197	-0.871	35.9	$[M-H]^-$	$[M-H]^-$	395.1331	1.180	20.6	$[M+H]^+$	-	L
hydroxytyrosol acyclodihydroelenolate	$C_{19}H_{26}O_8$	3.3	381.1560	0.482	22.1	$[M-H]^-$, $[M-H_2O-H]^-$	[M-H]-, $[M-H-H_2O]$-	365.1599	-1.575	47.2	$[M-H_2O+H]^+$, $[M+H]^+$	-	L
oleuropein	$C_{25}H_{32}O_{13}$	3.4	539.1769	-0.116	11.1	$[M-H]^-$	[M-H]-, [M+Cl]-	523.1764	-4.748	53.6	$[M-H_2O+H]^+$, $[M+H]^+$	$[M-H_2O+H]^+$, $[M+H]^+$	S
azelaic acid	$C_9H_{16}O_4$	3.4	187.0971	-0.535	2.6	$[M-H]^-$, $[M-H_2O-H]^-$	$[M-H]^-$	189.1125	-1.125	7.7	$[M+H]^+$, $[M+Na]^+$	-	T

Compound	Neutral Molecular formula	Rt (min)	Negative					Positive					ID
			m/z	Error (mDa)	mSigma	ESI MS signal	APCI MS signal	*m/z*	Error (mDa)	mSigma	ESI MS signal	APCI MS signal	
acetylated hydroxytyrosol	$C_{10}H_{12}O_4$	3.4	195.0658	-0.522	8.2	$[M-H]^-$, $[M-H_2O-H]^-$	$[M-H]^-$	197.0814	0.588	14.2	$[M+H]^+$, $[M-H_2O+H]^+$, $[M+K]^+$, $[M+Na]^+$	-	I
hydroxypinoresinol	$C_{20}H_{22}O_7$	3.6	373.1294	0.079	7.3	$[M-H]^-$	$[M-H]^-$	375.1451	0.479	17.8	$[M+H]^+$	-	L
hydroxydecarboxymethyloleuropein aglycone	$C_{17}H_{20}O_7$	3.6	335.1135	-0.183	18.3	$[M-H]^-$	$[M-H]^-$	337.1275	-0.700	19.1	$[M+H]^+$	-	L
elenolic acid	$C_{11}H_{14}O_6$	3.6	241.0720	0.271	1.7	$[M-H]^-$	$[M-H]^-$	225.0765	0.716	10	$[M-H_2O+H]^+$, $[M+H]^+$, $[M+K]^+$, $[M+Na]^+$	$[M-H_2O+H]^+$, $[M+H]^+$	I
dihydrokaempferol	$C_{15}H_{12}O_6$	3.6	287.0565	0.337	11.3	$[M-H]^-$	[M-H]-, [M-H-H_2O]-	289.0708	-0.230	23.6	$[M+H]^+$, $[M-H_2O+H]^+$	$[M+H]^+$	Met Frag/ SL
lucidumoside C	$C_{27}H_{36}O_{14}$	3.7	583.2031	-0.235	2.8	$[M-H]^-$	$[M-H]^-$	-	-	-	-	-	L
ligstroside	$C_{25}H_{32}O_{12}$	3.9	523.1820	-0.179	3.5	$[M-H]^-$	$[M-H]^-$	507.1852	-0.900	15.9	$[M-H_2O+H]^+$	-	L
luteolin	$C_{15}H_{10}O_6$	4.2	285.0405	0.136	2.4	$[M-H]^-$	$[M-H]^-$	287.0557	0.723	2.9	$[M+H]^+$	$[M+H]^+$	S
quercetin	$C_{15}H_{10}O_7$	4.3	301.0351	-0.194	17	$[M-H]^-$	$[M-H]^-$	303.0506	-0.923	34.5	$[M+H]^+$	$[M+H]^+$	S
hydroxydecanoic acid	$C_{10}H_{20}O_3$	4.3	187.1338	0.187	0.4	$[M-H]^-$	$[M-H]^-$	171.1379	-0.312	28.3	$[M-H_2O+H]^+$, $[M+H]^+$, $[M+Na]^+$	-	T
decarboxymethyloleuropein aglycone	$C_{17}H_{20}O_6$	4.4	319.1187	-0.018	9.9	$[M-H]^-$	$[M-H]^-$	321.1338	0.718	27.2	$[M+H]^+$	-	I
oleuropein aglycone (six isomers)	$C_{19}H_{22}O_8$	4.4, 4.6, 5.7, 5.8, 6.2, 7.5	377.1242	0.069	3.1	$[M-H]^-$	[M-H]-, [M-H-H_2O]-	379.1387	-0.095	4.3	$[M+H]^+$, $[M-H_2O+H]^+$	$[M+H]^+$	I
syringaresinol	$C_{22}H_{26}O_8$	4.4	417.1551	-2.794	29.3	$[M-H]^-$	-	419.1691	-0.944	29.6	$[M-H_2O+H]^+$	-	L
elenolic acid methylester	$C_{12}H_{16}O_6$	4.5	255.0876	-0.273	12.4	$[M-H]^-$	-	257.1022	0.407	38.1	$[M-H_2O+H]^+$, $[M+H]^+$	-	L
pinoresinol	$C_{20}H_{22}O_6$	4.7	357.1337	-0.625	20.7	$[M-H]^-$	$[M-H]^-$	359.1489	-4.095	17.6	$[M+H]^+$	$[M+H]^+$	S
t-cinnamic acid	$C_9H_8O_2$	4.8	147.0447	-0.772	15.6	$[M-H]^-$	$[M-H]^-$	131.0485	0.060	12.5	$[M-H_2O+H]^+$, $[M+H]^+$	-	S
acetoxypinoresinol	$C_{22}H_{24}O_8$	4.9	415.1404	-1.632	8.2	$[M-H]^-$	-	417.1539	-0.515	15.2	$[M+H]^+$	-	I
trihydroxyoctadecadienoic acid	$C_{18}H_{32}O_5$	5.0	327.2180	0.034	6.9	$[M-H]^-$	$[M-H]^-$	329.2330	-0.216	15.9	[M+H]+	-	T
trihydroxyoctadecenoic acid	$C_{18}H_{34}O_5$	5.1	329.2335	0.129	16.9	$[M-H]^-$, $[M-H_2O-H]^-$	$[M-H]^-$	331.2490	1.077	17.2	$[M+H]^+$, $[M+K]^+$	$[M+H]^+$, $[M-H_2O+H]^+$	L
decarboxymethylligstroside aglycone	$C_{17}H_{20}O_5$	5.1	303.1238	0.177	5.3	[M-H]-, $[M-H_2O-H]^-$	[M-H]-	305.1394	0.167	11.3	$[M+H]^+$, $[M-H_2O+H]^+$, $[M+K]^+$, $[M+Na]^+$	-	I
naringenin	$C_{15}H_{12}O_5$	5.1	271.0613	0.086	13.4	$[M-H]^-$	$[M-H]^-$	273.0760	-2.464	28.7	$[M+H]^+$	$[M+H]^+$	SL
apigenin	$C_{15}H_{10}O_5$	5.1	269.0459	0.329	13.3	$[M-H]^-$	$[M-H]^-$	271.0605	0.398	4.5	$[M+H]^+$	$[M+H]^+$	S
unknown 5	$C_{21}H_{26}O_9$	5.4	421.1507	0.088	5.8	$[M-H]^-$	$[M-H]^-$	423.1631	0.684	28.1	$[M+H]^+$	-	-
diosmetin	$C_{16}H_{12}O_6$	5.4	299.0558	-0.280	14.9	$[M-H]^-$	$[M-H]^-$	301.0714	0.711	2.5	$[M+H]^+$	$[M+H]^+$	L
hydroxydecarboxymethylligstroside aglycone	$C_{17}H_{20}O_6$	5.4	319.1189	-0.417	16.8	$[M-H]^-$, $[M-H_2O-H]^-$	$[M-H]^-$	321.1343	0.984	22.3	$[M+H]^+$	-	L
trihydroxyoctadecanoic acid	$C_{18}H_{36}O_5$	5.4	331.2488	-0.139	6.5	$[M-H]^-$	$[M-H]^-$	333.2641	-0.026	17.3	$[M+H]^+$, $[M-H_2O+H]^+$	-	L
ligstroside aglycone (six isomers)	$C_{19}H_{22}O_7$	5.4, 5.7, 5.8, 7.1, 7.3, 8.3	361.1293	-0.010	3.4	$[M-H]^-$	$[M-H]^-$	363.1443	0.501	6.4	$[M+H]^+$, $[M-H_2O+H]^+$	$[M+H]^+$	I

Compound	Neutral Molecular formula	Rt (min)	Negative					Positive					ID
			m/z	Error (mDa)	mSigma	ESI MS signal	APCI MS signal	*m/z*	Error (mDa)	mSigma	ESI MS signal	APCI MS signal	
methyldecarboxymethyloleuropein aglycone	$C_{18}H_{22}O_6$	5.8	333.1343	-0.018	4.9	$[M-H]^-$	$[M-H]^-$	317.1385	1.817	11.9	$[M-H_2O+H]^+$	-	L
dehydrooleuropein aglycone	$C_{19}H_{20}O_8$	5.8	375.1087	0.164	21.7	$[M-H]^-$	$[M-H]^-$	377.1243	2.783	18.3	$[M+H]^+$	$[M+H]^+$	L
dihydroxyhexadecanoic acid	$C_{16}H_{32}O_4$	6.1	287.2230	0.184	1.4	$[M-H]^-$	$[M-H]^-$	289.2384	1.106	13.6	$[M+H]^+$, $[M-H_2O+H]^+$, $[M+Na]^+$	-	L
methyloleuropein aglycone	$C_{20}H_{24}O_8$	6.1	391.1395	0.239	12.6	$[M-H]^-$	$[M-H]^-$	393.1537	-0.841	24.2	$[M+H]^+$	-	L
dehydroligstroside aglycone	$C_{19}H_{20}O_7$	7.0	359.1138	-0.071	19.9	$[M-H]^-$	$[M-H]^-$	361.1294	1.211	25.1	$[M+H]^+$	-	L
gingerol	$C_{17}H_{26}O_4$	8.0	293.1759	0.114	4.2	$[M-H]^-$	$[M-H]^-$	295.1906	0.324	35.6	$[M+H]^+$	-	Met Frag
monohydroxylated derivative of maslinic acid	$C_{30}H_{48}O_5$	8.0	487.3429	-0.020	4.8	$[M-H]^-$	$[M-H]^-$	489.3536	-1.358	19.2	$[M+H]^+$	-	L
dimethyloleuropein aglycone	$C_{21}H_{26}O_8$	8.2	405.1558	-0.235	16.3	$[M-H]^-$	$[M-H]^-$	407.1695	0.096	32.3	$[M+H]^+$	-	L
unknown 6	$C_{20}H_{38}O_5$	8.2	357.2641	-0.567	12.1	$[M-H]^-$	$[M-H]^-$	359.2794	-0.084	21.2	$[M+H]^+$, $[M-H_2O+H]^+$, $[M+Na]^+$	-	-
unknown 7	$C_{25}H_{36}O_7$	8.2	447.2388	-0.054	30	$[M-H]^-$	$[M-H]^-$	449.2514	-3.351	17.1	$[M+H]^+$	-	-
unknown 8	$C_{26}H_{38}O_7$	8.4	461.2545	-0.029	1.5	$[M-H]^-$	$[M-H]^-$	463.2789	3.377	28.6	$[M+H]^+$	$[M+H]^+$	-
unknown 9	$C_{25}H_{36}O_6$	8.6	431.2448	0.128	3.4	$[M-H]^-$	$[M-H]^-$	433.2571	0.366	24.3	$[M+H]^+$, $[M+Na]^+$	$[M+H]^+$	-
hydroxyoctadecatrienoic acid	$C_{18}H_{30}O_3$	8.6	293.2122	-0.027	14.5	$[M-H]^-$	$[M-H]^-$	295.2278	0.007	17.4	$[M+H]^+$	$[M+H]^+$, $[M-H_2O+H]^+$	T
dihydroxyoctadecanoic acid	$C_{18}H_{36}O_4$	8.7	315.2516	-2.789	13.9	$[M-H]^-$	$[M-H]^-$	317.2692	0.338	16.4	$[M+H]^+$, $[M-H_2O+H]^+$	-	T
hydroxyoctadecadienoic acid	$C_{18}H_{32}O_3$	8.7	295.2277	-0.131	15.2	$[M-H]^-$, $[M-H_2O-H]^-$	$[M-H]^-$	297.2429	-1.337	28.5	$[M+H]^+$	$[M+H]^+$	T
dihydroxyoctadecadienoic acid	$C_{18}H_{32}O_4$	8.8	311.2222	-0.241	14.1	$[M-H]^-$	$[M-H]^-$	313.2378	-0.348	15.2	$[M+H]^+$	-	T
hydroxyoctadecenoic acid	$C_{18}H_{34}O_3$	8.9	297.2435	-0.062	17.2	$[M-H]^-$	$[M-H]^-$	299.2592	0.297	36.8	$[M+H]^+$	$[M+H]^+$	T
maslinic acid	$C_{30}H_{48}O_4$	8.9	471.3488	0.783	15.4	$[M-H]^-$	$[M-H]^-$	455.3503	-1.355	11.9	$[M-H_2O+H]^+$, $[M+H]^+$	$[M+H]^+$, $[M-H_2O+H]^+$	S
hydroxyoctadecanoic acid	$C_{18}H_{36}O_3$	8.8	299.2591	-0.071	1.5	$[M-H]^-$	$[M-H]^-$	301.2742	0.479	23.3	$[M+H]^+$	-	T
linolenic acid	$C_{18}H_{30}O_2$	9.1	277.2174	0.122	13.9	$[M-H]^-$	$[M-H]^-$	279.2321	-0.385	18.5	$[M+H]^+$, $[M-H_2O+H]^+$	$[M+H]^+$	S
betulinic acid	$C_{30}H_{48}O_3$	9.4	455.3538	0.568	6.4	$[M-H]^-$	$[M-H]^-$	457.3668	-0.822	8.3	$[M+H]^+$	$[M+H]^+$	S
palmitoleic acid	$C_{16}H_{30}O_2$	9.6	253.2178	0.427	8.7	$[M-H]^-$	$[M-H]^-$	277.2151	0.677	19.7	$[M+Na]^+$, $[M+H]^+$, $[M-H_2O+H]^+$	$[M+H]^+$, $[M-H_2O+H]^+$	S
oleanolic acid	$C_{30}H_{48}O_3$	9.6	455.3537	-0.314	5.9	$[M-H]^-$	$[M-H]^-$	457.3669	-0.722	5.3	$[M+H]^+$	$[M+H]^+$	S
linoleic acid	$C_{18}H_{32}O_2$	9.7	279.2333	0.469	2.3	$[M-H]^-$	$[M-H]^-$	281.2481	0.526	17.8	$[M+H]^+$	$[M-H_2O+H]^+$, $[M+H]^+$	S
hydroxyeicosanoic acid	$C_{20}H_{40}O_3$	9.9	327.2907	-0.726	18.5	$[M-H]^-$	$[M-H]^-$	329.3050	0.360	14.1	$[M+H]^+$	-	T
palmitic acid	$C_{16}H_{32}O_2$	9.9	255.2333	0.345	10.9	$[M-H]^-$	$[M-H]^-$	257.2475	0.558	13.5	$[M+H]^+$	-	SL
oleic acid	$C_{18}H_{34}O_2$	10.0	281.2497	0.173	12.4	$[M-H]^-$	$[M-H]^-$	283.2638	0.915	19.2	$[M+H]^+$	$[M+H]^+$, $[M-H_2O+H]^+$	S
erythrodiol	$C_{30}H_{50}O_2$	10.2	-	-	-	-	-	443.3868	-0.866	11.9	$[M+H]^+$	$[M+H]^+$	S
uvaol	$C_{30}H_{50}O_2$	11.2	-	-	-	-	-	443.3868	-1.359	15.3	$[M+H]^+$	$[M+H]^+$	S
stearic acid	$C_{18}H_{36}O_2$	10.6	283.2642	0.509	10.2	$[M-H]^-$	$[M-H]^-$	285.2801	-0.340	26.4	$[M+H]^+$	$[M+H]^+$	L
lupeol isomers I and II	$C_{30}H_{50}O$	11.6, 11.9	-	-	-	-	-	409.3835	-0.655	10.3	$[M-H_2O+H]^+$, $[M+H]^+$	$[M-H_2O+H]^+$	L

Compound	Neutral Molecular formula	Rt (min)	Negative					Positive					ID
			m/z	Error (mDa)	mSigma	ESI MS signal	APCI MS signal	*m/z*	Error (mDa)	mSigma	ESI MS signal	APCI MS signal	
δ-tocopherol	$C_{27}H_{46}O_2$	12.1	-	-	-	-	-	401.3401	-1.307	25.3	$[M+H-H_2]^-$	$[M+H-H_2]^-$	S
β+γ-tocopherol	$C_{28}H_{48}O_2$	12.7	432.3606	-	-	?	?	415.3563	-0.800	15.6	$[M+H-H_2]^-$	$[M+H-H_2]^-$	S
cycloartenol	$C_{30}H_{50}O$	12.8	-	-	-	-	-	409.3828	0.082	19.5	$[M-H_2O+H]^+$, $[M+H]^+$	$[M-H_2O+H]^+$	L
stigmastadienol	$C_{29}H_{48}O$	13.2	-	-	-	-	-	395.3658	1.184	27.5	-	$[M-H_2O+H]^+$	L
a-tocopherol	$C_{29}H_{50}O_2$	13.4	446.3756	-	-	?	?	429.3713	-1.400	9.3	$[M+H-H_2]^-$	$[M+H-H_2]^-$	S
Δ^5-avenasterol	$C_{29}H_{48}O$	13.6	-	-	-	-	-	395.3676	-0.316	7.9	$[M-H_2O+H]^+$	$[M-H_2O+H]^+$	L
stigmasterol	$C_{29}H_{48}O$	13.8	-	-	-	-	-	395.3661	1.107	20.5	-	$[M-H_2O+H]^+$	S
campesterol	$C_{28}H_{48}O$	14.4	-	-	-	-	-	383.3679	-0.690	11.1	$[M-H_2O+H]^+$	$[M-H_2O+H]^+$	S
citrostadienol	$C_{30}H_{50}O$	14.6	-	-	-	-	-	409.3830	-0.164	9.2	-	$[M-H_2O+H]^+$	L
methylencycloartanol	$C_{31}H_{52}O$	15.2	-	-	-	-	-	423.3977	-0.169	15.9	$[M-H_2O+H]^+$	$[M-H_2O+H]^+$	L
β-sitosterol	$C_{29}H_{50}O$	15.3	-	-	-	-	-	397.3836	-0.715	29.4	-	$[M-H_2O+H]^+$	S

m/z, error and mSigma correspond to the first mentioned signal in the ESI column (when available); adducts are sorted by relative abundance.
Different isomers have been included within the same line of the table, indicating the corresponding Rt of each one. They all have been considered for giving the global numbers regarding annotated compounds.
Meaning of abbreviations used in the ID column: S, standard; I, isolated fraction; SL, spectral library; L, previously reported in literature; T, tentative annotation (previous reports missing).

Table S2. List of compounds detected with GC-APCI-MS.

Compound	M	Rt	*m/z*	Error (mDa)	mSigma	Calculated molecular formula	Signal	Other MS signals						ID
unknown 1	$C_{11}H_{18}O_8$	6.9	**351.1475**	0.430	36.3	$C_{14}H_{27}O_8Si$	[M-H+TMS+H]$^+$	261.0981	($C_{11}H_{17}O_7$)					-
unknown 2	$C_8H_{10}O_3$	7.1	227.1117	-0.851	17.6	$C_{11}H_{19}O_3Si$	[M-H+TMS+H]$^+$	**137.0630**	($C_8H_9O_2$)					-
unknown 3	$C_8H_{12}O_3$	7.6	**139.0786**	-0.507	14.4	$C_8H_{11}O_2$	[M-H+TMS-OTMS+H]$^+$							-
vanillin	$C_8H_8O_3$	8	**225.0942**	-0.203	15.6	$C_{11}H_{15}O_3Si$	[M-H+TMS+H]$^+$	209.0635	($C_{10}H_{13}O_3Si$)					S
t-cinnamic acid	$C_9H_8O_2$	8.1	221.0987	-0.862	30.9	$C_{12}H_{17}O_2Si$	[M-H+TMS+H]$^+$	**205.0649**	($C_{11}H_{13}O_2Si$)					S
tyrosol*	$C_8H_{10}O_2$	8.8	282.1466	-1.205	36.2	$C_{14}H_{26}O_2Si_2$	[M-2H+2TMS]$^+$	**193.1058**	($C_{11}H_{17}OSi$)	179.0894	($C_{10}H_{15}OSi$)			S
4-hydroxybenzoic acid	$C_7H_6O_3$	9.9	283.1184	-0.009	17.2	$C_{13}H_{23}O_3Si_2$	[M-2H+2TMS+H]$^+$							S
unknown 4	$C_9H_{14}O_3$	11.4, 12.2	**243.1409**	-0.170	31.6	$C_{12}H_{23}O_3Si$	[M-H+TMS+H]$^+$	153.0935	($C_9H_{13}O_2$)					-
vanillic acid	$C_8H_8O_4$	13.6	**313.1278**	-0.463	28.8	$C_{14}H_{25}O_4Si_2$	[M-2H+2TMS+H]$^+$	223.0643	($C_{11}H_{15}O_3Si$)	297.0940	($C_{13}H_{21}O_4Si_2$)			S
hydroxytyrosol*	$C_8H_{10}O_3$	13.7	370.1809	-0.699	21	$C_{17}H_{34}O_3Si_3$	[M-3H+3TMS]$^+$	**281.1392**	($C_{14}H_{25}O_2Si_2$)	193.0688	($C_{11}H_{17}OSi$)			S
acetylated hydroxytyrosol*	$C_{10}H_{12}O_4$	14.9	340.1523	-0.703	32	$C_{16}H_{28}O_4Si_2$	[M-2H+2TMS]$^+$	**281.1389**	($C_{14}H_{25}O_2Si_2$)					I
unknown 5	$C_{13}H_{20}O_7$	15.0/16.0/17.3/19.5	**361.1685**	-0.221	29.1	$C_{16}H_{28}O_7Si$	[M-H+TMS+H]$^+$							-
unknown 6	$C_{12}H_{18}O_8$	15.3	**363.1480**	-0.999	22.3	$C_{16}H_{28}O_7Si$	[M-H+TMS+H]$^+$	273.0982	($C_{12}H_{16}O_7$)					-
elenolic acid isomer I	$C_{11}H_{14}O_6$	15.4	315.1256	-0.276	1.1	$C_{14}H_{23}O_6Si$	[M-H+TMS+H]$^+$	**225.0768**	($C_{11}H_{13}O_5$)	283.1002	($C_{13}H_{19}O_5Si$)			I
elenolic acid isomer II	$C_{11}H_{14}O_6$	16	315.1258	-0.155	5.6	$C_{14}H_{23}O_6Si$	[M-H+TMS+H]$^+$	**225.0771**	($C_{11}H_{13}O_5$)	283.0999	($C_{13}H_{19}O_5Si$)			I
unknown 7	$C_{14}H_{24}O_9$	16.5	**553.2686**	-0.664	17.3	$C_{23}H_{49}O_9Si_3$	[M-3H+3TMS+H]$^+$	373.1682	($C_{17}H_{29}O_7Si$)	283.1189	($C_{14}H_{19}O_6$)			-
quinic acid	$C_7H_{12}O_6$	16.6	**481.2286**	-0.434	15.3	$C_{19}H_{45}O_6Si_4$	[M-4H+4TMS+H]$^+$	391.1783	($C_{16}H_{35}O_5Si_3$)	301.1273	($C_{13}H_{25}O_4Si_2$)	211.0741	($C_{10}H_{15}O_3Si$)	S
unknown 8	$C_6H_{10}O_6$	17.0, 17.3	**467.2132**	-0.093	8.6	$C_{18}H_{43}O_6Si_4$	[M-4H+4TMS+H]$^+$							-
p-coumaric acid	$C_9H_8O_3$	17.7	**309.1333**	-0.392	4.1	$C_{15}H_{25}O_3Si_2$	[M-2H+2TMS+H]$^+$	237.0910	($C_{12}H_{17}O_3Si$)	381.1728	($C_{18}H_{33}O_3Si_3$)	293.1012	($C_{14}H_{21}O_3Si_2$)	S
unknown 9	$C_{20}H_{38}O_{12}$	17.8, 18.2	**543.2837**	-0.598	17.4	$C_{23}H_{47}O_{12}Si$	[M-H+TMS+H]$^+$	453.2339	($C_{20}H_{37}O_{11}$)					-
elenolic acid isomer III	$C_{11}H_{14}O_6$	17.9	315.1256	-0.188	8.3	$C_{14}H_{23}O_6Si$	[M-H+TMS+H]$^+$	**225.0769**	($C_{11}H_{13}O_5$)	283.0995	($C_{13}H_{19}O_5Si$)			I
palmitoleic acid	$C_{16}H_{30}O_2$	19.7	**327.2712**	-0.193	40.1	$C_{19}H_{39}O_2Si$	[M-H+TMS+H]$^+$							S
unknown 10	$C_{13}H_{24}O_{10}$	20	629.3185	-0.063	53.2	$C_{25}H_{57}O_{10}Si_4$	[M-4H+4TMS+H]$^+$	**539.2671**	($C_{22}H_{47}O_9Si_3$)					-
palmitic acid	$C_{16}H_{32}O_2$	20.1	**329.2868**	-0.287	40.8	$C_{19}H_{41}O_2Si$	[M-H+TMS+H]$^+$							L
ferulic acid	$C_{10}H_{10}O_4$	21.3	**339.1439**	0.153	7.2	$C_{16}H_{27}O_4Si_2$	[M-2H+2TMS+H]$^+$	411.1832	($C_{19}H_{35}O_4Si_3$)	249.0915	($C_{13}H_{17}O_3Si$)			S
linoleic acid	$C_{18}H_{32}O_2$	23.8	**353.2867**	-0.248	4.6	$C_{21}H_{41}O_2Si$	[M-H+TMS+H]$^+$	263.2347	($C_{18}H_{31}O$)					S
oleic acid	$C_{18}H_{34}O_2$	23.9	**355.3023**	-0.432	3.6	$C_{21}H_{43}O_2Si$	[M-H+TMS+H]$^+$	265.2525	($C_{18}H_{33}O$)					S
linolenic acid	$C_{18}H_{30}O_2$	24	**351.2711**	-0.318	43.5	$C_{21}H_{39}O_2Si$	[M-H+TMS+H]$^+$	261.2213	($C_{18}H_{29}O$)					S
stearic acid	$C_{18}H_{36}O_2$	24.4	**357.3170**	-2.597	47.9	$C_{21}H_{45}O_2Si$	[M-H+TMS+H]$^+$	267.2684	($C_{18}H_{35}O$)					L
arachidic acid	$C_{20}H_{40}O_2$	28.5	**385.3490**	-1.145	41.6	$C_{23}H_{49}O_2Si$	[M-H+TMS+H]$^+$	295.2996	($C_{20}H_{39}O$)					L
decarboxymethylligstroside aglycone	$C_{17}H_{20}O_5$	30.4	377.1773	-0.104	7.5	$C_{20}H_{29}O_5Si$	[M-H+TMS+H]$^+$	359.1669	($C_{20}H_{27}O_4Si$)	**193.1056**	($C_{11}H_{17}OSi$)			I
dihydroxyhexadecanoic acid	$C_{16}H_{32}O_4$	31.1	**415.3054**	0.415	19.4	$C_{22}H_{47}O_3Si_2$	[M-3H+3TMS-OTMS+H]$^+$	505.3546	($C_{25}H_{57}O_4Si_3$)	325.2558	($C_{19}H_{37}O_2Si$)			T
decarboxymethyloleuropein aglycone	$C_{17}H_{20}O_6$	33.7	465.2110	-0.139	11.4	$C_{23}H_{37}O_6Si_2$	[M-2H+2TMS+H]$^+$	375.1620	($C_{20}H_{27}O_5Si$)	**193.1057**	($C_{11}H_{17}OSi$)	281.1386	($C_{14}H_{25}O_2Si_2$)	I
glyceryl linoleate	$C_{21}H_{38}O_4$	33.9, 34.5	427.3236	-0.864	18.6	$C_{24}H_{47}O_4Si$	[M-H+TMS+H]$^+$	501.3777	($C_{27}H_{57}O_4Si_2$)	**411.3282**	($C_{24}H_{47}O_3Si$)			L

Compound	M	Rt	*m/z*	Error (mDa)	mSigma	Calculated molecular formula	Signal	Other MS signals						ID
ligstroside aglycone isomer I	$C_{19}H_{22}O_7$	35.4	435.1832	-0.184	7.6	$C_{22}H_{31}O_7Si$	$[M-H+TMS+H]^+$	475.1959	($C_{24}H_{35}O_6Si_2$)	**193.1058**	($C_{11}H_{17}OSi$)			I
squalene	$C_{30}H_{50}$	35.5	**411.3978**	0.74	18.8	$C_{30}H_{51}$	$[M+H]^+$							L
ligstroside aglycone isomer II	$C_{19}H_{22}O_7$	36.7	507.2225	-0.553	9.6	$C_{25}H_{39}O_7Si_2$	$[M-2H+2TMS+H]^+$	475.1957	($C_{24}H_{35}O_6Si_2$)	**193.1059**	($C_{11}H_{17}OSi$)			I
δ-tocopherol	$C_{27}H_{46}O_2$	36.9	**475.3958**	-0.808	15.8	$C_{30}H_{55}O_2Si$	$[M-H+TMS+H]^+$							S
ligstroside aglycone isomer III	$C_{19}H_{22}O_7$	37.6	507.2224	-0.538	5.8	$C_{25}H_{39}O_7Si_2$	$[M-2H+2TMS+H]^+$	**193.1058**	($C_{11}H_{17}OSi$)					I
oleuropein aglycone isomer I	$C_{19}H_{22}O_8$	38.2	523.2176	-0.132	9.5	$C_{25}H_{39}O_8Si_2$	$[M-2H+2TMS+H]^+$	**281.1384**	($C_{14}H_{25}O_2Si_2$)					I
β-tocopherol	$C_{28}H_{48}O_2$	38.3	**489.4107**	-1.72	12	$C_{31}H_{57}O_2Si$	$[M-H+TMS+H]^+$							S
γ-tocopherol	$C_{28}H_{48}O_2$	38.5	**489.4108**	-1.627	9.1	$C_{31}H_{57}O_2Si$	$[M-H+TMS+H]^+$							S
oleuropein aglycone isomer II	$C_{19}H_{22}O_8$	38.7	523.2176	-0.223	4.8	$C_{25}H_{39}O_2Si_2$	$[M-2H+2TMS+H]^+$	**281.1384**	($C_{14}H_{25}O_2Si_2$)					I
oleuropein aglycone isomer III	$C_{19}H_{22}O_8$	39.4	595.2568	-0.259	6.6	$C_{28}H_{47}O_8Si_3$	$[M-3H+3TMS+H]^+$	**281.1389**	($C_{14}H_{25}O_2Si_2$)					I
apigenin isomer	$C_{15}H_{10}O_5$	39.7	415.1387	-0.471	37.4	$C_{21}H_{27}O_5Si_2$	$[M-2H+2TMS+H]^+$							T
hydroxyoleuropein aglycone	$C_{19}H_{22}O_9$	39.8	611.2517	-0.834	19.7	$C_{28}H_{47}OSi_3$	$[M-3H+3TMS+H]^+$	**281.1387**	($C_{14}H_{25}O_2Si_2$)					L
oleuropein aglycone isomer IV	$C_{19}H_{22}O_8$	40.2	595.2572	-0.495	7.5	$C_{28}H_{47}O_8Si_3$	$[M-3H+3TMS+H]^+$	**281.1385**	($C_{14}H_{25}O_2Si_2$)					I
α-tocopherol	$C_{29}H_{50}O_2$	40.7	**503.4254**	-2.48	15.2	$C_{32}H_{59}O_2Si$	$[M-H+TMS+H]^+$							S
apigenin	$C_{15}H_{10}O_5$	40.8	**487.1780**	-0.577	4.2	$C_{24}H_{35}O_5Si_3$	$[M-3H+3TMS+H]^+$	415.1389	($C_{21}H_{27}O_5Si_2$)	471.1467	($C_{23}H_{31}O_5Si_3$)			S
luteolin isomer	$C_{15}H_{10}O_6$	42.3	503.1727	-0.961	8.2	$C_{24}H_{35}O_6Si_3$	$[M-3H+3TMS+H]^+$							T
campesterol	$C_{28}H_{48}O$	42.4	473.4147	-2.608	12.9	$C_{31}H_{57}OSi$	$[M-H+TMS+H]^+$	**383.3666**	($C_{28}H_{47}$)					S
stigmasterol	$C_{29}H_{48}O$	42.9	485.4160	-1.339	6.3	$C_{32}H_{57}OSi$	$[M-H+TMS+H]^+$	**395.3669**	($C_{29}H_{47}$)					S
luteolin	$C_{15}H_{10}O_6$	43.1	**575.2124**	-0.871	6.2	$C_{27}H_{43}O_6Si_4$	$[M-4H+4TMS+H]^+$	503.1726	($C_{24}H_{35}O_6Si_3$)	559.1801	($C_{26}H_{39}O_6Si_4$)			S
pinoresinol	$C_{20}H_{22}O_6$	43.7	503.2262	-1.69	18.1	$C_{26}H_{39}O_6Si_2$	$[M-2H+2TMS+H]^+$	**485.2165**	($C_{26}H_{37}O_5Si_2$)					S
β-sitosterol	$C_{29}H_{50}O$	43.8	487.4318	-1.675	25.1	$C_{32}H_{59}OSi$	$[M-H+TMS+H]^+$	**397.3819**	($C_{29}H_{49}$)					S
Δ^5-avenasterol	$C_{29}H_{48}O$	44	485.4159	-1.361	8.4	$C_{32}H_{57}OSi$	$[M-H+TMS+H]^+$	**395.3656**	($C_{29}H_{47}$)					L
acetoxypinoresinol*	$C_{22}H_{24}O_8$	44.3	560.2255	-0.629	38.03	$C_{28}H_{40}O_8Si_2$	$[M-2H+2TMS]^+$	501.21117	($C_{26}H_{37}O_6Si_2$)					I
Δ^5-stigmastadienol	$C_{29}H_{48}O$	44.5	485.4162	-1.284	12.7	$C_{32}H_{57}OSi$	$[M-H+TMS+H]^+$	**395.3656**	($C_{29}H_{47}$)					L
cycloartenol	$C_{30}H_{50}O$	44.6	499.4318	-1.3	6.3	$C_{33}H_{59}OSi$	$[M-H+TMS+H]^+$	**409.3817**	($C_{30}H_{49}$)					L
methylencycloartanol	$C_{31}H_{52}O$	45.5	513.4473	-1.269	6.3	$C_{34}H_{61}OSi$	$[M-H+TMS+H]^+$	**423.3977**	($C_{31}H_{51}$)					L
eythrodiol	$C_{30}H_{50}O_2$	46.1	497.4163	-1.099	40.7	$C_{33}H_{57}OSi$	$[M-H_2O-H+TMS+H]^+$	**407.3680**	($C_{30}H_{47}$)					S
citrostadienol	$C_{30}H_{50}O$	46.1	499.4314	-1.549	6.8	$C_{33}H_{59}OSi$	$[M-H+TMS+H]^+$	**409.4382**	($C_{30}H_{49}$)					L
uvaol	$C_{30}H_{50}O_2$	46.6	497.4165	-0.801	35.6	$C_{33}H_{57}OSi$	$[M-H_2O-H+TMS+H]^+$	**407.3671**	($C_{30}H_{47}$)					S
oleanolic acid	$C_{30}H_{48}O_3$	47	**511.3961**	-0.489	8.1	$C_{33}H_{55}O_2Si$	$[M-H+TMS]^+$	601.4452	($C_{36}H_{65}O_3Si_2$)					S
betulinic acid	$C_{30}H_{48}O_3$	47.2	**601.4448**	-1.823	4.3	$C_{36}H_{65}O_3Si_2$	$[M-2H+2TMS]^+$	511.3954	($C_{33}H_{55}O_2Si$)					S
ursolic acid	$C_{30}H_{48}O_3$	47.6	**511.3958**	-0.781	18.8	$C_{33}H_{55}O_2Si$	$[M-H+TMS]^+$	601.4471	($C_{36}H_{65}O_3Si_2$)					S
maslinic acid I	$C_{30}H_{48}O_4$	48	617.4401	-0.492	15.7	$C_{36}H_{65}O_4Si_2$	$[M-2H+2TMS]^+$	**527.3902**	($C_{33}H_{55}O_3Si$)	509.3799	($C_{33}H_{53}O_2Si$)	599.4296	($C_{36}H_{63}O_3Si_2$)	S
maslinic acid II	$C_{30}H_{48}O_4$	49.4	527.3909	-0.541	13.3	$C_{33}H_{55}O_3Si$	$[M-H+TMS+H]^+$	**509.3799**	($C_{33}H_{53}O_2Si$)	599.4296	($C_{36}H_{63}O_3Si_2$)			S

* Compounds detected as $[M-nH+nTMS]^+$. MS Signals with the highest relative abundance are presented in bold letters.
In this table, the isomers are included by using different lines, since in some cases the achieved MS information was slightly different.
Meaning of abbreviations used in the ID column: S, standard; I, isolated fraction; L, previously reported in literature; T, tentative annotation (previous reports missing).

Table S3. Distribution of the determined metabolites in the eight evaluated samples (all the given values are % referred to the richest sample regarding each analyte).

	Leaves	Wood	Skin	Pulp	VOO	Dehydro VOO	Seed	Seed Oil
gallic acid	100	64	0	0	0	1	0	0
protocatechuic acid	3	6	100	1	0	1	0	0
eudesmic acid	10	9	14	10	73	100	11	66
gentisic acid	0	56	0	0	0	100	0	0
4-hydroxybenzoic acid	100	31	19	20	0	0	0	0
4-hydroxyphenylacetic acid	100	48	24	69	12	7	10	1
vanillic acid	0	14	100	12	77	37	0	19
syringic acid	0	0	97	0	0	100	0	0
homovanillic acid	0	0	100	59	31	43	0	24
vanillin	0	0	100	0	56	76	0	42
caffeic acid	18	85	67	100	0	0	38	19
p-coumaric acid	23	4	100	22	64	70	5	3
verbascoside	89	100	0	107	0	0	14	0
sinapic acid	0	0	0	0	0	100	0	79
ferulic acid	27	40	16	44	100	77	4	25
t-cinnamic acid	0	0	0	0	12	100	12	72
Phenolic acids and aldehydes	56	38	100	44	67	84	12	48
quinic acid	61	62	46	100	8	1	1	0
citric acid	57	100	49	76	0	0	96	0
aesculin	2	100	0	1	0	0	0	0
aesculetin	2	100	1	1	0	0	0	0
Organic acids and coumarins	62	100	49	94	5	0	40	0
3,4-dihydroxyphenylglycol	32	16	26	47	100	11	14	0
oxydized hydroxytyrosol	73	54	53	100	36	5	18	0
hydroxytyrosol glucoside	25	100	3	95	0	1	33	0
hydroxytyrosol	29	43	5	100	7	20	14	3
tyrosol glucoside	6	44	7	100	0	1	72	0
tyrosol	8	3	33	58	91	100	6	23
2-phenethyl β-primeveroside	100	34	3	32	1	0	12	0
acetylated hydroxytyrosol	0	100	80	0	25	87	4	21
gingerol	45	77	82	69	90	100	89	66
Simple phenols and derivatives	31	83	10	100	8	12	39	4
hydroxydecarboxymethylelenolic acid	42	3	82	100	8	17	12	0
acyclodihydroelenolic acid hexoside	24	16	47	100	1	0	22	0
decarboxylated form of hydroxy elenolic acid	15	7	100	65	0	74	10	9
dihydrooleuropein	3	10	0	100	0	0	1	0
oleoside/secologanoside	82	100	39	45	0	0	4	0
elenolic acid glucoside	31	24	16	100	0	1	28	0
decarboxymethylelenolic acid	63	71	100	51	93	64	2	51
hydroxyoleuropein	85	100	4	22	0	0	15	0
oleuropein glucoside	48	66	0	41	0	0	100	0
hydroxyelenolic acid	2	1	100	36	11	45	1	0
nuzhenide	1	1	1	4	0	0	100	2
cafselogoside	1	3	1	100	0	0	3	0
desoxyelenolic acid	11	3	3	30	27	5	100	18
comselogoside	1	0	6	100	1	0	1	0
10-hydroxyoleuropein aglycone	100	27	11	79	12	2	0	0
hydroxytyrosol acyclodihydroelenolate	0	0	0	100	0	0	0	0
oleuropein	46	100	1	61	0	0	8	0

	Leaves	Wood	Skin	Pulp	VOO	Dehydro VOO	Seed	Seed Oil
hydroxydecarboxymethyloleuropein aglycone	6	100	21	48	42	59	1	0
elenolic acid	9	1	5	100	11	40	1	1
lucidumoside C	100	71	2	27	0	0	32	0
ligstroside	30	100	11	0	0	1	97	0
decarboxymethyloleuropein aglycone	52	100	4	98	86	55	1	0
elenolic acid methylester	23	5	76	54	17	100	0	0
oleuropein aglycone	16	17	0	100	6	1	0	0
decarboxymethylligstroside aglycone	9	4	0	9	100	35	1	0
hydroxydecarboxymethylligstroside aglycone	100	10	0	58	11	91	0	0
ligstroside aglycone	12	10	1	100	79	14	5	1
methyldecarboxymethyloleuropein aglycone	16	18	4	67	100	5	2	1
dehydrooleuropein aglycone	0	34	2	84	100	16	3	1
methyloleuropein aglycone	0	72	21	100	0	19	6	0
dehydroligstroside aglycone	0	0	0	14	100	31	3	3
dimethyloleuropein aglycone	11	14	0	100	0	0	0	0
Secoiridoids and derivatives	28	31	8	100	20	14	10	1
luteolin diglucoside	100	0	0	0	0	0	0	0
cyanidin 3-O-glucoside	0	100	0	0	0	0	1	0
gallocatechin	100	30	3	13	0	0	17	0
rutin	100	76	2	23	0	0	1	0
luteolin 7-O-rutinoside	100	8	8	23	0	0	0	0
quercetin 4'-O-glucoside	9	100	0	0	0	0	0	0
luteolin 7-O-glucoside	100	34	9	22	0	0	0	0
apigenin 7-O-rutinoside	100	7	13	12	0	0	1	0
luteolin 4'-O-glucoside	100	19	4	4	0	0	0	0
taxifolin	15	100	0	0	0	0	0	0
apigenin 7-O-glucoside	100	21	14	20	0	0	0	0
chrysoeriol 7-O-glucoside	100	4	1	0	0	0	0	0
luteolin glucoside isomer	100	13	8	0	0	0	0	0
dihydrokaempferol	0	100	0	0	0	0	0	0
luteolin	100	16	91	29	46	5	0	0
quercetin	46	100	7	1	4	1	0	0
naringenin	3	100	7	1	28	4	0	1
apigenin	15	5	39	7	100	16	1	0
diosmetin	100	13	34	1	97	19	1	0
Flavonoids	100	72	24	15	22	3	7	0
hydroxypinoresinol	1	4	100	1	7	5	0	2
syringaresinol	11	22	100	12	70	68	10	34
pinoresinol	4	10	100	1	61	29	7	23
acetoxypinoresinol	0	0	68	0	65	0	23	100
Lignans	2	6	100	1	21	12	2	9
azelaic acid	1	0	100	0	2	2	0	4
hydroxydecanoic acid	0	0	7	0	0	0	0	100
trihydroxyoctadecadienoic acid	4	2	100	0	1	0	1	0
trihydroxyoctadecenoic acid	0	0	100	0	0	1	0	0
trihydroxyoctadecanoic acid	0	0	100	0	0	1	0	0
dihydroxyhexadecanoic acid	1	0	100	0	0	2	0	0
hydroxyoctadecatrienoic acid	65	13	60	1	97	71	5	100
dihydroxyoctadecanoic acid	1	1	21	1	1	1	1	100
hydroxyoctadecadienoic acid	2	1	100	1	26	27	4	70

	Leaves	Wood	Skin	Pulp	VOO	Dehydro VOO	Seed	Seed Oil
dihydroxyoctadecadienoic acid	0	0	66	1	6	15	1	100
hydroxyoctadecenoic acid	1	3	0	1	10	11	2	100
hydroxyoctadecanoic acid	8	15	4	27	88	13	19	100
linolenic acid	19	10	11	6	100	67	7	26
palmitoleic acid	4	4	0	8	70	100	9	61
linoleic acid	0	1	100	6	15	5	5	2
hydroxyeicosanoic acid	3	1	0	1	29	29	1	100
palmitic acid	64	100	0	55	18	7	52	10
oleic acid	27	29	0	100	65	12	65	11
stearic acid	0	100	0	79	0	0	44	0
Fatty acids and derivatives	1	1	100	1	4	5	1	21
monohydroxylated derivative of maslinic acid	31	5	100	1	4	9	1	8
maslinic acid	10	6	100	0	1	8	0	1
betulinic acid	38	100	4	0	1	9	0	1
oleanolic acid	45	23	100	0	1	19	0	1
uvaol & erythrodiol	47	32	100	0	90	46	0	1
Pentacyclic triterpenes	22	13	100	0	2	12	0	1
δ-tocopherol	0	0	0	0	32	65	0	100
β- & γ-tocopherol	16	0	0	19	61	89	26	100
α-tocopherol	72	11	0	50	100	33	8	88
Tocopherols	67	10	0	48	100	44	12	96
lupeol	0	0	0	0	17	100	0	0
lupeol isomer	0	0	0	0	16	100	0	0
cycloartenol	0	0	0	58	35	100	0	0
stigmastadienol	0	0	0	0	29	100	0	0
Δ^5-avenasterol	6	0	0	21	42	100	2	20
stigmasterol	0	0	0	0	57	100	0	0
campesterol	0	0	4	10	22	28	16	100
citrostadienol	0	0	0	0	6	36	8	100
methylencycloartanol	0	0	0	17	19	100	0	14
β-sitosterol	13	10	5	23	25	76	25	100
Sterols	10	7	4	21	30	100	19	89

Each chemical class was determined in the most favorable coupling (maximum number of identified compound and good ionization rate avoiding saturation in any matrix): organic acids, coumarins and phenolic compounds (phenolic acids and aldehydes, simple phenols, secoiridoids, flavonoids and lignans) in LC-ESI-MS$^{(-)}$; fatty acids and derivatives as well as triterpenic acids in LC-APCI-MS$^{(-)}$; and triterpenic alcohols, tocopherols and sterols in LC-APCI-MS$^{(+)}$.
